# Functional characterization of GABA_A_ receptor-mediated modulation of cortical neuron network activity in microelectrode array recordings

**DOI:** 10.1371/journal.pone.0186147

**Published:** 2017-10-13

**Authors:** Benjamin M. Bader, Anne Steder, Anders Bue Klein, Bente Frølund, Olaf H. U. Schroeder, Anders A. Jensen

**Affiliations:** 1 NeuroProof GmbH, Friedrich-Barnewitz-Str. 4, Rostock, Germany; 2 Department of Drug Design and Pharmacology, Faculty of Health and Medical Sciences, University of Copenhagen, Universitetsparken 2, Copenhagen Ø, Denmark; Georgia State University, UNITED STATES

## Abstract

The numerous γ-aminobutyric acid type A receptor (GABA_A_R) subtypes are differentially expressed and mediate distinct functions at neuronal level. In this study we have investigated GABA_A_R-mediated modulation of the spontaneous activity patterns of primary neuronal networks from murine frontal cortex by characterizing the effects induced by a wide selection of pharmacological tools at a plethora of activity parameters in microelectrode array (MEA) recordings. The basic characteristics of the primary cortical neurons used in the recordings were studied in some detail, and the expression levels of various GABA_A_R subunits were investigated by western blotting and RT-qPCR. In the MEA recordings, the pan-GABA_A_R agonist muscimol and the GABA_B_R agonist baclofen were observed to mediate phenotypically distinct changes in cortical network activity. Selective augmentation of αβγ GABA_A_R signaling by diazepam and of δ-containing GABA_A_R (δ-GABA_A_R) signaling by DS1 produced pronounced changes in the majority of the activity parameters, both drugs mediating similar patterns of activity changes as muscimol. The apparent importance of δ-GABA_A_R signaling for network activity was largely corroborated by the effects induced by the functionally selective δ-GABA_A_R agonists THIP and Thio-THIP, whereas the δ-GABA_A_R selective potentiator DS2 only mediated modest effects on network activity, even when co-applied with low THIP concentrations. Interestingly, diazepam exhibited dramatically right-shifted concentration-response relationships at many of the activity parameters when co-applied with a trace concentration of DS1 compared to when applied alone. In contrast, the potencies and efficacies displayed by DS1 at the networks were not substantially altered by the concomitant presence of diazepam. In conclusion, the holistic nature of the information extractable from the MEA recordings offers interesting insights into the contributions of various GABA_A_R subtypes/subgroups to cortical network activity and the putative functional interplay between these receptors in these neurons.

## Introduction

γ-Aminobutyric acid (GABA), the major inhibitory neurotransmitter in the central nervous system (CNS), exerts its physiological effects through two distinct receptor families, the GABA_A_ and GABA_B_ receptors (GABA_A_Rs and GABA_B_Rs, respectively) [1–3]. The GABA_A_Rs are a highly heterogenous family of ligand-gated anion-selective channels, and the abundance of GABAergic neurons and the key roles played by GABA_A_Rs in the CNS means that the receptors hold considerable potential as targets for therapeutic invention in a wide range of central disorders. Thus, GABA_A_Rs are targeted by drugs used in the clinical treatment of sleeping disorders, anxiety and epilepsy, and they are pursued as putative drug targets in numerous neurodegenerative, cognitive and psychiatric disorders [4–9]. However, the widespread expression of GABA_A_Rs and the multiple functions governed by different receptor subtypes are also believed to be at the root of many of the adverse effects associated with the fairly non-selective GABA_A_R-based therapeutics currently available. Thus, an increased understanding of the physiological functions mediated by different GABA_A_Rs could potentially form the basis for development of more efficient and/or safer drugs [2, 3].

The GABA_A_R is a pentameric membrane-bound subunit complex, and the heterogeneity of native GABA_A_R populations arises from the existence of a total of 19 subunits (α_1_-α_6_, β_1_-β_3_, γ_1_-γ_3_, δ, ε, π, θ, ρ_1_-ρ_3_) that assemble into a plethora of receptor subtypes differentially expressed throughout the CNS [2, 3]. The molecular diversity of GABA_A_Rs is also considerable at the neuronal level. The synaptic GABA_A_Rs expressed at postsynaptic densities that mediate “phasic inhibition” are predominantly αβγ receptors composed of α_1_, α_2_ and/or α_3_, β_2_ and/or β_3_, and γ subunits (typically γ_2_) [2, 3]. In addition to these receptors, activation of perisynaptic or extrasynaptic GABA_A_Rs by low levels of ambient GABA gives rise to a persistent “tonic inhibition” that regulates the excitability and the firing mode of the neuron [10, 11]. The α_4_βδ receptors constitute the major extrasynaptic GABA_A_R in most brain regions, in particular in the forebrain, with α_6_βδ GABA_A_Rs being the predominant extrasynaptic receptors in cerebellum and with α_5_βγ_2_ and α_1_βδ receptors being important mediators of tonic inhibition in hippocampal and neocortical pyramidal cells and interneurons, respectively [2, 10–13]. These different locations of the receptors at the neuronal body mean that they mediate different contributions to synaptic transmission and neuronal activity. Both overall excitability and output of the specific neuron and global neuronal network activity is regulated by a multitude of mechanisms, and a component of the homeostatic plasticity of GABAergic synapses seems to arise from yet poorly elucidated interactions between phasic and tonic inhibition [14].

The respective roles of different GABA_A_R subtypes in GABAergic neurotransmission have most often been studied in electrophysiological recordings of phasic and tonic currents in cultured neurons or brain slices from rodents. While these recordings provide detailed insight into the GABA_A_Rs expressed in specific neurons and their importance for neuronal activity and synaptic transmission, the recordings do not necessarily elucidate the overall effects of GABA_A_R-mediated signaling at entire neuronal networks. The use of microelectrode arrays (MEAs) enables the recordings of extracellular action potentials of cultured neurons and thus elucidatation of the activity characteristics of neuronal networks. For more than 20 years, this technological platform has been used for studying neurotoxic effects of compounds and toxins [15–17] but also in studies investigating the functional modes of action of ligands [17–22]. In the present study, we have applied this technology to study the effects induced by a broad selection of pharmacological tools characterized by different GABA_A_R subtype-selectivity profiles at the spontaneous activity patterns of primary neuronal networks from murine frontal cortex, and in this way we have elucidated GABA_A_R-mediated effects on neuronal activity in a more holistic manner than possible by conventional slice and patch-clamp electrophysiology.

## Materials and methods

### Drugs

THIP (4,5,6,7-tetrahydroisoxazolo[5,4-*c*]pyridin-3-ol, also termed gaboxadol) and Thio-THIP (4,5,6,7-tetrahydroisothiazolo[5,4-c]pyridin-3-ol) were synthesized in-house essentially as previously described [23, 24]. DS1 (4-chloro-N-[6,8-dibromo-2-(2-thienyl)imidazo[1,2-a]pyridine-3-yl benzamide), DS2 (4-chloro-N-[2-(2-thienyl)imidazo[1,2-a]pyridine-3-yl benzamide) and flunitrazepam were obtained from Sigma-Aldrich (Copenhagen, Denmark), Tocris Cookson (Bristol, UK) and Roche (Mannheim, Germany), respectively, and diazepam, clonazepam, zolpidem, muscimol and baclofen were purchased from Sigma-Aldrich (Munich, Germany). The *in vitro* pharmacological properties of the respective drugs are given in Table 1 and will be outlined given in more detail in *Results*.

**Table 1:** Pharmacological properties of the drugs used in the present study.

Drug	Pharmacological properties
Baclofen	pan-GABA_B_R agonist
Muscimol	pan-GABA_A_R agonist
Diazepam	Benzodiazepine, PAM at α_1,2,3,5_βγ GABA_A_Rs
Flunitrazepam	Benzodiazepine, PAM at α_1,2,3,5_βγ GABA_A_Rs
Clonazepam	Benzodiazepine, PAM at α_1,2,3,5_βγ GABA_A_Rs
Zolpidem	Benzodiazepine-site modulator, PAM at α_1,2,3,5_βγ GABA_A_Rs, exhibits potency-based preference for α_1_βγ GABA_A_Rs
THIP	GABA_A_R agonist, exhibits potency-based preference for δ-containing GABA_A_Rs
Thio-THIP	Functionally selective GABA_A_R ligand, exhibit agonism at α_4_β_1,3_δ and weak antagonism at αβγ and α_4_β_2_δ GABA_A_Rs
THIP	Allosteric agonist and PAM, efficacy-based selectivity for δ-containing GABA_A_Rs
Thio-THIP	PAM, efficacy-based selectivity for δ-containing GABA_A_Rs

### Ethics

All neural tissue from animal were prepared according to the EU Directive 2010/63/EU on the protection of animals used for scientific purposes (certification file number 7221.3–2). In this study no animal experiments were performed in accordance with the German Animal Protection §7/2 (Tierschutzgesetz). Time-pregnant animals were purchased and shipped by a licensed animal supplier Charles River, Germany. Animals were stored in a separate room for less than 24 hours after arrival in their transport boxes including food and water equivalent. Animal storage is supervised by an animal welfare officer at NeuroProof GmbH, Germany. Short-term storage of animals in transport boxes is in agreement with Directive (EG) Nr. 1/2005 (Animal safety during transport). The mice were sacrificed by cervical dislocation according to the German Animal Protection Act §4.

### Primary cell cultures

Pre-frontal cortex tissue was harvested from embryonic day 15/16 chr:NMRI mice (Charles River). Tissue was dissociated by enzymatic digestion (133,3 Kunitz units/ml DNase; 10 Units/ml papain) and mechanical trituration, counted, vitality controlled, and plated in DMEM containing 5% fetal bovine serum and 5% horse serum on poly-*D*-lysine- and laminin-coated microelectrode array (MEA) neurochips with 2x32 passive electrodes for two independent experiments per MEA (Center for Network NeuroScience, University of North Texas, Denton, TX). The density of plating was 4,000 cells per mm^2^. Cultures on the MEA chips were incubated at 37°C in a 10% CO_2_ atmosphere until ready for use, typically four weeks after seeding. Culture media was replenished twice a week with DMEM containing 10% horse serum. The developing co-cultures were treated with the mitosis inhibitor 5-fluoro-2'-deoxyuridine (25μM) and uridine (63 μM) for 48 h on day 5 after seeding to prevent further glial proliferation. After 4 weeks in culture, the activity pattern stabilizes and is composed of one coordinated main burst pattern with several coordinated sub-patterns [18, 19, 25–29]. In this study cultures between 28 and 35 days *in vitro* were used.

### Multichannel recordings and data analysis

Extracellular recordings were performed using a computer-controlled 64-channel MEA workstation acquisition system (Plexon, Inc., Dallas, TX), where temperature control of 37°C and stable pH of 7.4 (10% CO_2_) enabled stable recording and cumulative concentration-response determinations for periods longer than 10 h. Recordings were performed as previously reported using a computer-controlled 64-channel MEA workstation acquisition system (Plexon, Inc., Dallas, TX) providing amplification, filtering and digital signal processing of MEA signals [20]. The total system gain used was 10 K with a simultaneous 40 kHz sampling rate, and the signals routinely recorded by these neurochips are located in the range of 15–800 μV [20]. The neuronal networks were acutely treated with a series of accumulating increasing concentrations of the test compound (maximum assay concentration of DMSO: 0.1%), and the recordings were performed over time periodes of 8–9 h. The network response (spike rate) was observed online. Each of the test compound concentrations was applied and incubated for at least 60 minutes of which a stable phase of 30 minutes was used for data analysis, and real time unit separation and spike identification were performed in real time as previously described. The multichannel signal acquisition system delivered single neuron spike data including action potential waveforms. Spike identification and separation were accomplished using a template-matching algorithm in real time [20]. This permitted the simultaneous extracellular recording of action potentials from a maximum of 256 neurons per MEA, thus 128 per experiment. The action potentials, or “spikes”, were recorded as spike trains; they are clustered in so-called bursts. Bursts were quantitatively described via direct spike train analysis using the program NeuroEXplorer (Plexon Inc., Dallas, TX) and NPWaveX (NeuroProof GmbH, Rostock, Germany). Bursts were defined by the beginning and end of short spike events. Maximum spike intervals defining the start of a burst were adjusted from 50 to 150 ms, and maximum intervals to end a burst from 100 to 300 ms [20]. Bursts definition and high content analysis of the network activity patterns provided a multiparametric description characterizing the activity changes in four defined categories: “*General Activity*”, “*Burst Structure*”, “*Oscillatory Behaviour*” and “*Synchronicity*” (presented in detail in section 3.1.2). Unless otherwise specified, the parameters for each experiment and each experimental treatment were normalized to the corresponding values of the native reference activity. Our minimal acceptance criteria for active MEA networks was 6 bursting neurons in a network as this allows a network analysis using the synchronicity measures. In average 25 active units (from 32 electrodes) were recorded per network, with peaks of up to 100 units per network. The data from the recordings with DS2 (alone) has been published previously [22], whereas the other data presented in this study has not.

In the figures in this study, heat map presentations depicting the changes induced by the various pharmacological tools in 40 of the 204 activity parameters are given together with concentration-response curves for 10 selected activity parameters. The definitions of some of these activity parameters have been presented in a recent publication [30], and the definitions of all 40 parameters shown in the figures are given in S1 Table. In brief: *spike rate*: number of spikes per second, averaged over all spike trains recorded per 60 s bin; *burst rate*: number of bursts per second, a measure for burstiness of units averaged over all units recorded per 60 s bin; *burst duration*: mean lengths of bursts (in ms) based on inter-spike interval (ISI) method; *burst amplitude*: bursts are mathematically superimposed with an integral function defined by spike peak density in bursts and number of spikes, and burst amplitude is the peak amplitude of the integrated burst reflecting the fraction of the bursts with highest spike density; *spike rate SD and burst rate SD*: SD of spike and burst rate, respectively, across 60 s bins, indicating the variability of spikiness and burstiness of units within temporal episodes; *spike rate CVnet and burst rate CVnet*: coefficient of variation of spike and burst rate, respectively, reflecting spatial variation of spike and burst rate over the network during experimental episodes; *Syn All*: average distance of bursts within a population burst from population burst center, a measure for the strength of synchronicity of a network; *Spike Simplex;* spike trains are divided into timeframes of 1 ms bin-size, and within these bins different units within the network generate spikes. All units exhibiting a spike are defined as one simplex, and the outcome of the quantity of all simplex is the “Spike Simplex”, a measure for connectivity and complexity in neuronal network (higher values reflect higher synchronicity among neurons). Distribution of “Simplex events” are not distributed as in an *in silico*-generated poisson spike train. A detailed analysis shows that different units are involved in “Simplex events”. Doublete analyses (separation of units with identical origin) showed that the probability of doublets is below 1%.

### Statistical analysis

We used a minimum of 2 MEAs per animal preparation, and data were produced from cultures of at least 3 independent animal preparations. The data is pooled over all MEAs per test group. Concentration-response effects are shown as mean values ± SEM. Statistical analysis includes ANOVA followed by Student’s paired t-test when compared to native activity, unpaired t-test when compound effects are compared with each other. P values ≤ 0.05 are represented with *, p ≤ 0.01 with ** and p ≤ 0.001 with ***. Heat maps show only colored rectangles if p ≤ 0.05, colors encode effect sizes as shown in the color legend. The data for the different drugs are based on the following numbers of MEA experiments: DMSO: 12. muscimol: 7, baclofen: 8, diazepam: 11, clonazepam: 14, zolpidem: 12, flunitrazepam: 12, THIP: 9, Thio-THIP: 9, DS2: 11, DS1: 10, [DS2 + 3 μM THIP]: 17, [DS2 + 15 μM THIP]: 8, [THIP + 300 nM DS2]: 12, [THIP + 1 μM DS2]: 11, [THIP + 3 μM DS2]: 15, [diazepam + 30 nM DS1]: 9, [DS1 + 50 nM diazepam]: 14.

### Immunocytochemistry and microscopy

Primary prefrontal cortex neurons were fixed for 20 min with phosphate-buffered saline (PBS) containing 4% paraformaldehyd, and 4% sucrose, followed by blocking with PBS containing 40 nM NH_4_Cl. Membrane permeabilization was performed with PBS containing 0.1% Triton-X100 for 5 min. Permeabilized cells were blocked using PBS contining 1% BSA, 2% goat serum and 0.05% Tween20). Primary and secondary antibodies were incubated at room temparature for 1 h each in a humified chamber. The following antibodies and dilutions were used: α1 GABA_A_R (Synaptic Systems; 1:500), synapsin-1 (New England Biolabs; 1:200), β3-tubulin (Sigma, 1:1000), Hoechst-Bisbenzimid (Sigma Aldrich, 1 μg/ml), and secondary antibodies (1.500; Alexa fluor 488 and 594-conjugated goat-anti-mouse and goat-anti-rabbit, Invitrogen). Images were acquired using a Nikon Eclipse fluorescence microscope. Image analysis (synapse isolation) was performed using ImageJ software (NIH). Images were analyzed by semi-automatic image quantification tools (MS Excel macro-based in-house analyses; ImageJ, Rawak Software, NIH, USA). The following parameters were quantified per image, means and SEM’s were calculated afterwards: cell number: absolute number of nuclei / field (marker: nuclear staining, automatic counting after image processing (binary, watershed), neuronal number: absolute number of neuronal soma / field (marker: HuC/D-positive objects, manual and semi-automatic counting), synapse number: absolute number of synapse punctae / field (image binarized, automatic analysis: local automatic threshold setting, watershed filter for object separation).

### Western blotting

The primary cortical cells and mouse brains were lysed using RIPA lysis buffer. SDS gel electrophoresis was performed using 12% pre-cast Bis-Tris gels (Novex™, NuPAGE™). Immunoblotting was performed using iBlot System and transfer Stacks PVDF. All steps were performed according to the manufaturer’s instructions. 10 μg protein was loaded per lane. Membranes were blocked for 1 hour using WesternBreeze Blocker solution (all materials from Invitrogen). The following primary antibodies and dilutions were used: α1 GABA_A_R (Synaptic System, 1:375), α3 GABA_A_R (Millipore, 1:200), α5 GABA_A_R (Abd-Serotec, 1:200), PSD-95 (Cell Signalling, 1:1000) and SNAP-25 (Synaptic Systems, 1:5000). Two α2 GABA_A_R antibodies were tested but were found to lack specificity. Primary antibodies were incubated over night at 4°C. Secondary antibodies (goat-anti-mouse, goat-anti-rabbit, both AP-conjugated, Life Technologies) were incubated for 1 h at room temperature. AP-conjugate was incubate for 5 min before signal acquisition using BioRad ChemiDoc XRS System and Gen5 Software (BioRad) for band quantification. Data from at least 3 lysates were summarized for band quantification. Western blot data is shown as mean values ± S.D.

### RT-qPCR

Primary neurons were harvested on the same day as the MEA recordings were performed, and cDNA derived from mouse prefrontal cortex were used as control. Total RNA was extracted using RNA mini kit from Qiagen (Thermo Fisher Scientific, Waltham, MA) according to the manufacturer’s protocol. The reverse transcription was performed using qScript™ cDNA SuperMix (Quanta Biosciences, Gaithersburg, MD) on a standard PCR machine (25°C for 5 min, 42°C for 30 min, 85°C for 5 min) and cDNA stored at -20°C until further processing. qPCR was performed essentially as previously described in 96-well plates (Agilent Technologies, Santa Clara, CA) mixing PerfeCTa SYBR Green FastMix (Quanta Biosciences), nuclease free water (Qiagen, West Sussex, UK) and primers (TAG Copenhagen A/S (Copenhagen, Denmark) (Table 2) [31]. The Primer pairs were validated using a serial dilution of cDNA and only primers that showed single product amplification and efficiency of 100% (± 5%) were used for further analyses. The qPCR was performed with an initial denaturation step of 95°C for 30 s, followed by 40 cycles of 5 s at 95°C, 60°C for 15 seconds and 72°C for 10 s. To assure single-product amplification, a dissociation curve analysis was performed consisting of 60 s at 95°C, 30 s at 55°C and 30 s at 95°C. The qPCR was performed using the Agilent Mx3005P qPCR system (Agilent Technologies), and the corresponding MxPro software was used to determine the Ct values. The ΔCt values were calculated using 2^(Reference Ct—Target Ct)^ as previously described [32].

**Table 2:** Sequences of primers used in the RT-qPCR.

**Target Genes**	**Primers**
α_4_	F: 5’-AGAACTCAAAGGACGAGAAATTGT-3’ R: 5’-TTCACTTCTGTAACAGGACCCC-3’
β_1_	F: 5’- GGTTTGTTGTGCACACAGCTCC-3’ R: 5’- ATGCTGGCGACATCGATCCGC-3’
β_2_	F: 5’-GCTGGTGAGGAAATCTCGGTCCC-3’ R: 5’-CATGCGCACGGCGTACCAAA-3’
β_3_	F: 5’-GGGACCCCCGAAGTCGGGTCT-3’ R: 5’-GAGCGTAAACGACCCCGGGAA-3’
δ	F: 5’- TCAAATCGGCTGGCCAGTTCCC -3’ R: 5’- GCACGGCTGCCTGGCTAATCC -3’
**Reference gene**	**Primers**
Rpl13a	F: 5’- GGAGGGGCAGGTTCTGGTAT-3’ R: 5’- TGTTGATGCCTTCACAGCGT-3’

## Results

### MEA recordings and multi-parametric analysis of network activity in primary cortical neurons

In this study the effects of various pharmacological tools on network activity were characterized in MEA recordings at primary neurons from murine frontal cortex, and in our analysis of these data we took advantage of the high-content information provided by multichannel recordings. Following an outline of some basic characteristics of the primary cortical neurons used for the recordings and of the principles of the MEA recordings and the analysis of the data from them in this section, these results will be outlined in sections 3.2–3.6.

**Fig 1:**
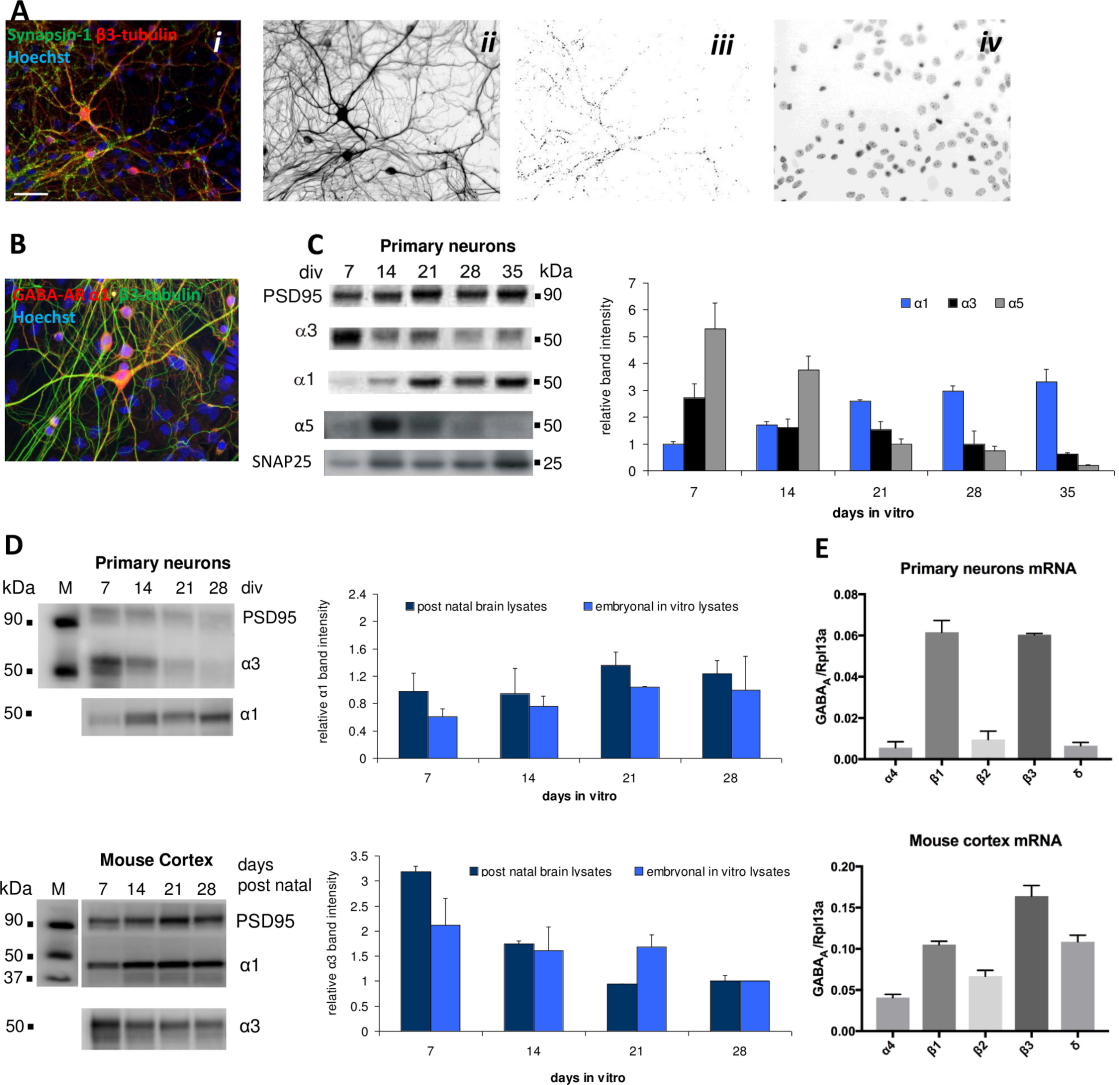
Biochemical characterization of cortical network characteristics and GABA_A_R subunit expression in primary cortical neurons and in mouse frontal cortex tissue. **A.** Immunocytochemistry characterization of the primary cortical neurons (28 DIV). *i*. Staining with antibodies for synapsin-1 (*green*), β3-tubulin (*red*) and Hoechst-Bisbenzimid (*blue*). *ii*. Neurons. *iii*. Isolated synaptic punctae. *iv*. Nuclei. **B.** Immunocytochemistry characterization of α_1_ GABA_A_R protein expression in the primary cortical neurons (28 DIV). Staining for α_1_ (*red*), β3-tubulin (*green*) and Hoechst-Bisbenzimid (*blue*). **C.** Western blot analysis of α_1_, α_3_ and α_5_ GABA_A_R protein expression levels in the primary neurons (7, 14, 21, 28 and 35 DIV). **D.** Western blot analysis of α_1_ and α_3_ protein expression levels in the primary neurons (7, 14, 21 and 28 DIV) and in postnatal mouse frontal cortex (7, 14, 21 and 28 days postnatally). **E.** RT-qPCR analysis of α_4_, β_1_, β_2_, β_3_ and δ mRNA expression levels (± S.D.) in the primary neurons and in postnatal mouse cortex tissue (relative to the expression of the reference gene RPL13a).

*Basic characteristics of the primary cortical neurons*. As mentioned in section 2.2, the primary neurons were used for the MEA recordings between 28 and 35 days *in vitro*. Due to the serum used in the culture medium glia survival is supported in these cultures, and mainly because of proliferation of glia during the first 4 days after plating these neuron-glia co-cultures thus consist of approximately 20% neurons and 80% astrocytes including 1% microglia [30]. This neuron/glia ratio of ~0.25 differs from the reported neuron/glia ratio of ~0.8 in mouse frontal cortex *in vivo* [33]. On the other hand, we have previously determined that approximately 20% of the neurons in these cultures are GABAergic neurons, which is in excellent agreement with ratio of GABAergic neurons (relative to total number of neurons) in mouse frontal cortex *in vivo* [30, 34]. In the present study, we investigated the composition of the cortical networks and the nature and degree of synapse formation in them by means of immunocytochemistry using antibodies targeting synapsin-1, β3-tubulin and Hoechst-Bisbenzimid as markers for synaptic punctae, neurons and nuclei, respectively (n = 6). As can be seen from Fig 1A, the structure and branching pattern of the neuronal networks looked comparable to those *in vivo*, and image analysis (synapse isolation) of cultures stained for synapsin-1 identified a high number of synaptic punctae per neuron. Although synapsin-1 is a neuron-specific phosphoprotein that binds selectively to vesicles in the presynaptic terminal and thus exclusively is a presynaptic marker [35], this nevertheless demonstrates the formation of a plethora of poly-synaptic connections in the cortical networks analogeously to *in vivo*.

Next we investigated to which extent the primary cortical neurons express the different GABA_A_R subtypes. Immunocytochemistry analysis using anti-α_1_ antibody demonstrated robust expression of this subunit at synaptic densities in the neurons (Fig 1B). Moreover, the expression levels of various GABA_A_R subunits in the primary neurons were investigated by western blotting and RT-qPCR analysis and compared to the expression levels of the subunits in postnatal mouse frontal cortex tissue. As can be seen from Fig 1C, developmental changes in expression of the α_1_, α_3_ and α_5_ proteins in the primary neurons at 7, 14, 21, 28 and 35 DIV differed, with α_1_ subunit levels increasing during culture while both α_3_ and α_5_ expression levels decreased over time. Importantly, these opposite trends for α_1_ and α_3_ expression levels over time were found to be comparable to the trends determined for the two subunits *in vivo* in lysates from frontal mouse cortex 7, 14, 21 and 28 days postnatally (Fig 1D), which again were in good agreement with the developmental trends for the two subunits reported from studies in rats and primates [36–39].

The expression levels of the α_4_ and δ GABA_A_R subunits in the primary neurons were also investigated by western blot analysis, and two different commercially available antibodies were tried for each subunit in these experiments. Despite these efforts we were unable to detect significant and convinving bands for either of these subunits when compared with the protein detected in control experiments performed on lysates from postnatal mouse frontal cortex. While these results certainly suggests that α_4_ and δ are expressed at lower levels than the α_1_, α_3_ and α_5_ subunits also studied in the western blotting experiments, the specificity and sensitivity of the antibodies used and/or the preparation of the lysate from the neuronal culture could also be contributing factors to the lack of detected bands for α_4_ and δ. Thus, the expression of α_4_ and δ in the primary neurons were investigated further by RT-qPCR analysis, where the respective expression levels of the mRNAs of the three β subunits also were determined. The α_4_ and δ transcripts were detected in similar levels in the neurons (Fig 1E). However, the expression levels of mRNAs for both subunits were substantially lower than the levels determined for the β_1_, β_2_ and β_3_ transcripts, with the levels of α_4_ and δ mRNAs being 11-, 1.7- and 11-fold and 9.3-, 1.5- and 9.2-fold lower, respectively, than those determined for β_1_, β_2_ and β_3_ (Fig 1E). For comparison, we also performed RT-qPCR analysis of postnatal mouse frontal cortex. It is important to stress that absolute mRNA levels of proteins in the primary neurons and in mouse frontal cortex are not directly comparable since the cellular composition of the tissue and the culture are very different, and thus the mRNA levels of α_4_, β_1_-β_3_ and δ in the tissue were as expected higher than the levels for the subunits in the primary neurons (Fig 1E). Interestingly, however, the relative expression of the five subunits followed roughly same pattern as seen in postnatal mouse frontal cortex, with a somewhat higher relative expression of δ compared being the major difference (Fig 1E). In conclusion, the expression levels of α_4_ and δ mRNAs in the primary neurons determined in the RT-qPCR analysis are sufficiently high to suggest that the subunits could be expressed at protein level, and importantly the lower expression levels of these two subunits compared to those of other α subunits and of the β subunits are in good agreement with the relative subunit expression levels in mouse frontal cortex. On the other hand, the apparently low expression levels of δ-GABA_A_Rs in the primary neurons used for the MEA recordings naturally raises the question, to which extent pharmacological modulation of δ-GABA_A_R signalling is capable of influencing network activity, which will be address further in the *Discussion* section.

**Fig 2:**
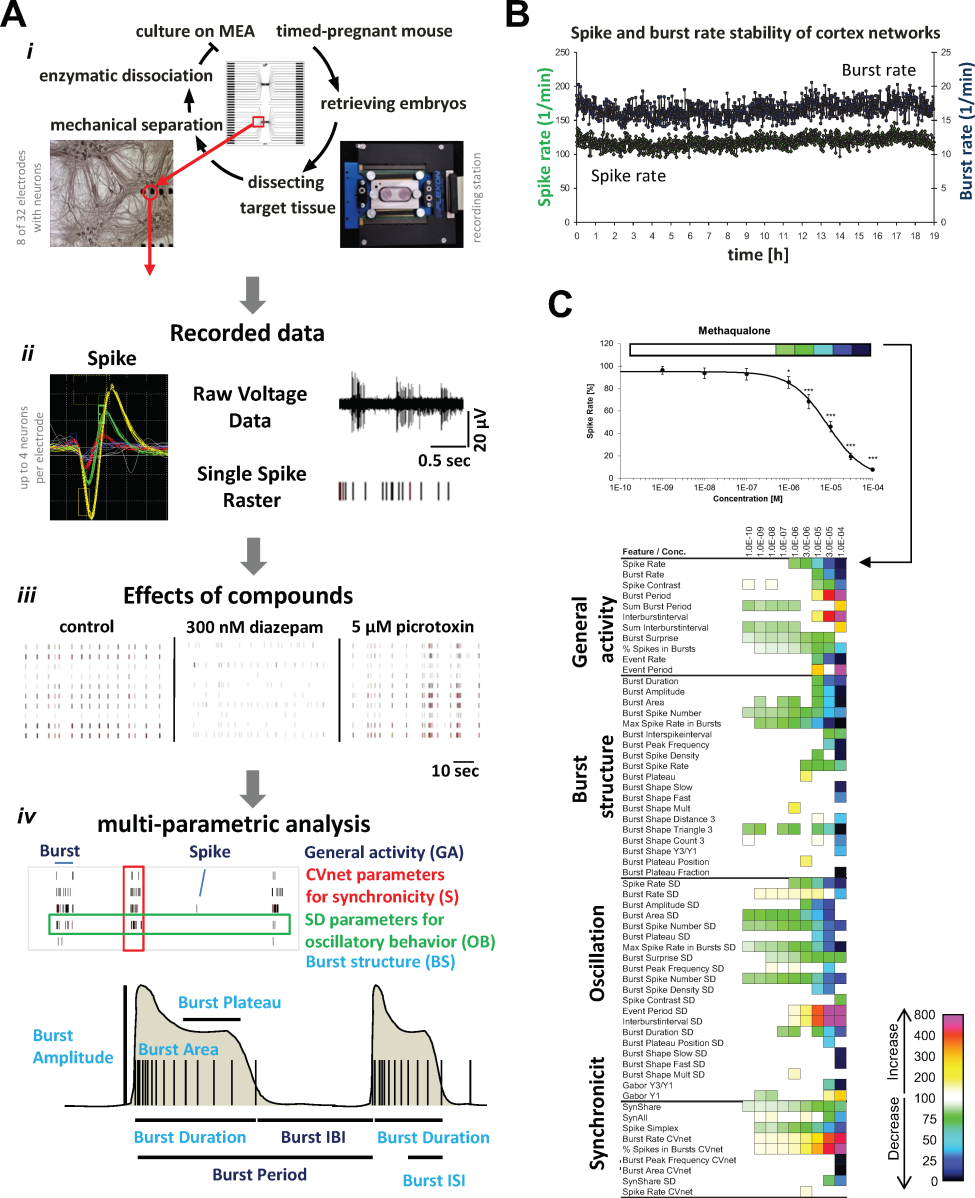
Multiparametric analysis of cortical neuron network activity. **A.** Recording setup and multiparametric analysis of cortical neuron network activity. *i*. Culture of neuronal networks on MEA neurochips. Two-well MEAs with 2x32 electrodes were used to acquire extra-cellular action potentials called spikes. *ii*. Examples of raw data from MEA recordings at cortical neurons. Wave forms from different neurons are separated yielding high-quality spike train data of single neurons in a network. *iii*. Spike raster plots of native cortical activity (control) and cortical activity after acute treatment with 300 nM diazepam or 5 μM picrotoxin. The augmentation and inhibition of GABA_A_R signaling mediated by the two respective drugs is observed to reduce and increase overall spiking and bursting activity, respectively. *iv*. Multiparametric analysis of MEA data. Top: Raw data (magnified detail from a spike raster plot, *iii*) with the origin of the four defined categories of activity parameters indicated. Bottom: Scheme of two simplified bursts outlining some of the major *General Activity* [burst inter burst interval (IBI) and burst period] and *Burst Structure* [burst duration, burst plateau, burst amplitude, burst inter-spike interval (ISI) and burst area] parameters extractable from the recordings. **B.** Stability of spike rate and burst rate of cortical networks recorded over 19 h in the absence of drug. **C.** Exemplification of the data presention in Figs 3–10. The effects of cumulatively increasing concentrations of the GABA_A_R PAM methaqualone on network activity parameters are given as concentration-response curve for the “Spike Rate” parameter (*top*) and as a heat map depicting statistically significant changes in several activity parameters relative to native activity (the activity at time point 0, 100%, Student’s paired t-test, p≤0.05) (*bottom*). The methaqualone data has been published previously [22].

*MEA recordings and multi-parametric analysis of network activity*. Spike rate is a parameter describing general activity but this is not nessecarily the most descriptive parameter that can be derived from MEA recordings. Thus, multi-parametric data analysis has in recent years gained more attention as spatio-temporal recordings of action potentials from numerous individual neurons offer additional insights into network functionality [17]. In addition to information about the effects of a substance on spike rates and burst rates, information about its impact on synchronicity, regularity of oscillation, burst structure and network connectivity can also be derived by this analysis (Fig 2A). In particular burst structure characteristics are essential features for describing the changes in the activity patterns induced by a substance. Moreover, analysis of synchronicity is possible with a high resolution in time and per neuron, and oscillatory behaviour can be measured as the rates of action potentials occurring with periodicity over time (Fig 2A). Both synchronicity and oscillation are important characteristic phenomena in neuronal network activity *in vitro* [17, 20, 22]. We observed this synchronized and regular activitiy pattern also *in vivo* using multi-electrode intra-cortical recordings which underlines the physiological relevance of the in vitro patterns (in-house data).

**Fig 3:**
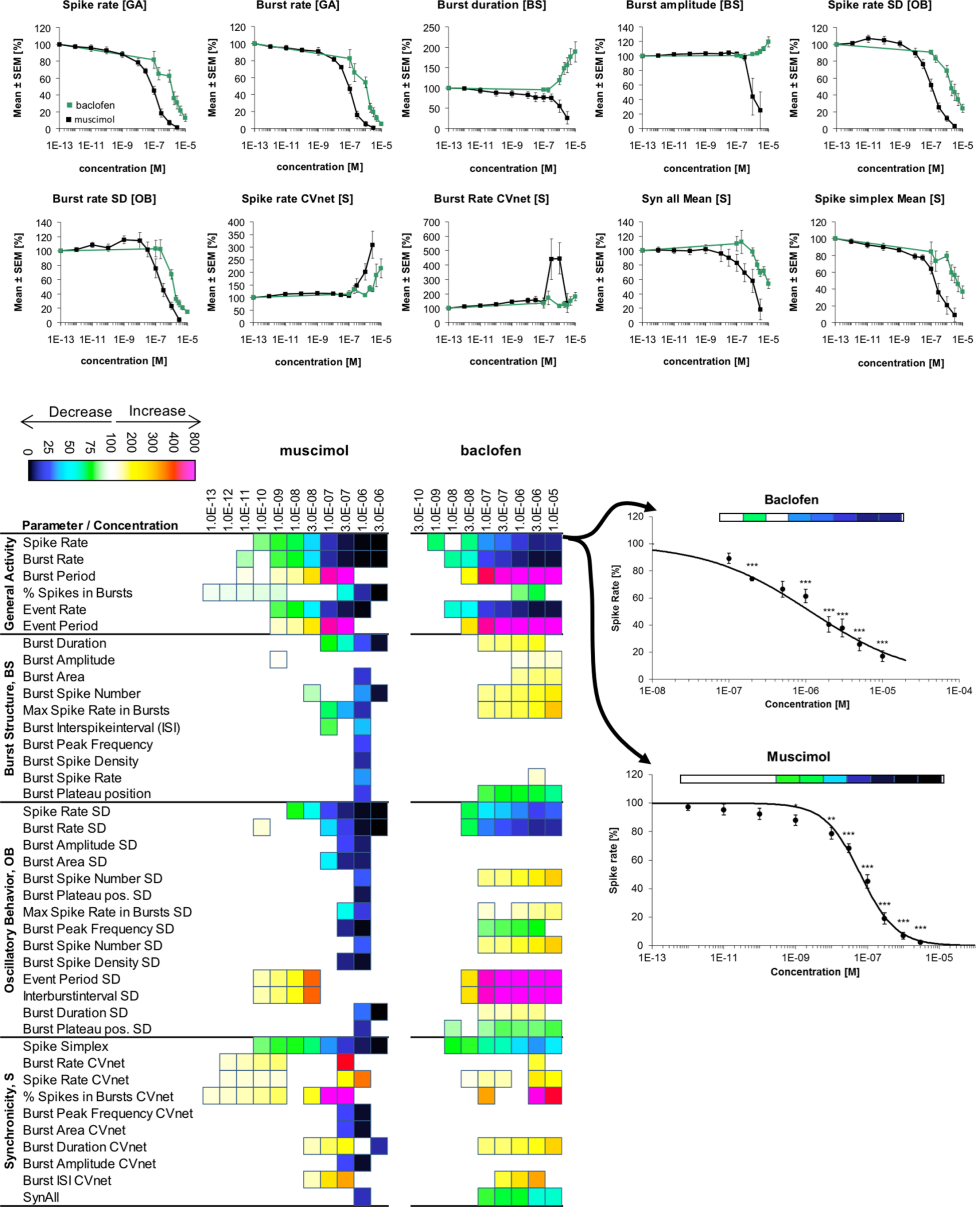
Changes in cortical network activity induced by 7–10 cumulatively increasing concentrations of the GABA_A_R agonist muscimol and the GABA_B_R agonist baclofen. The heat maps present statistically significant changes in 40 activity parameters relative to native activity (no drug, 100%) (Student’s paired t-test, p≤0.05). The concentration-response relationships for the drugs at 10 selected activity parameters are given as mean ± S.E.M. relative to native activity (no drug, 100%).

**Fig 4:**
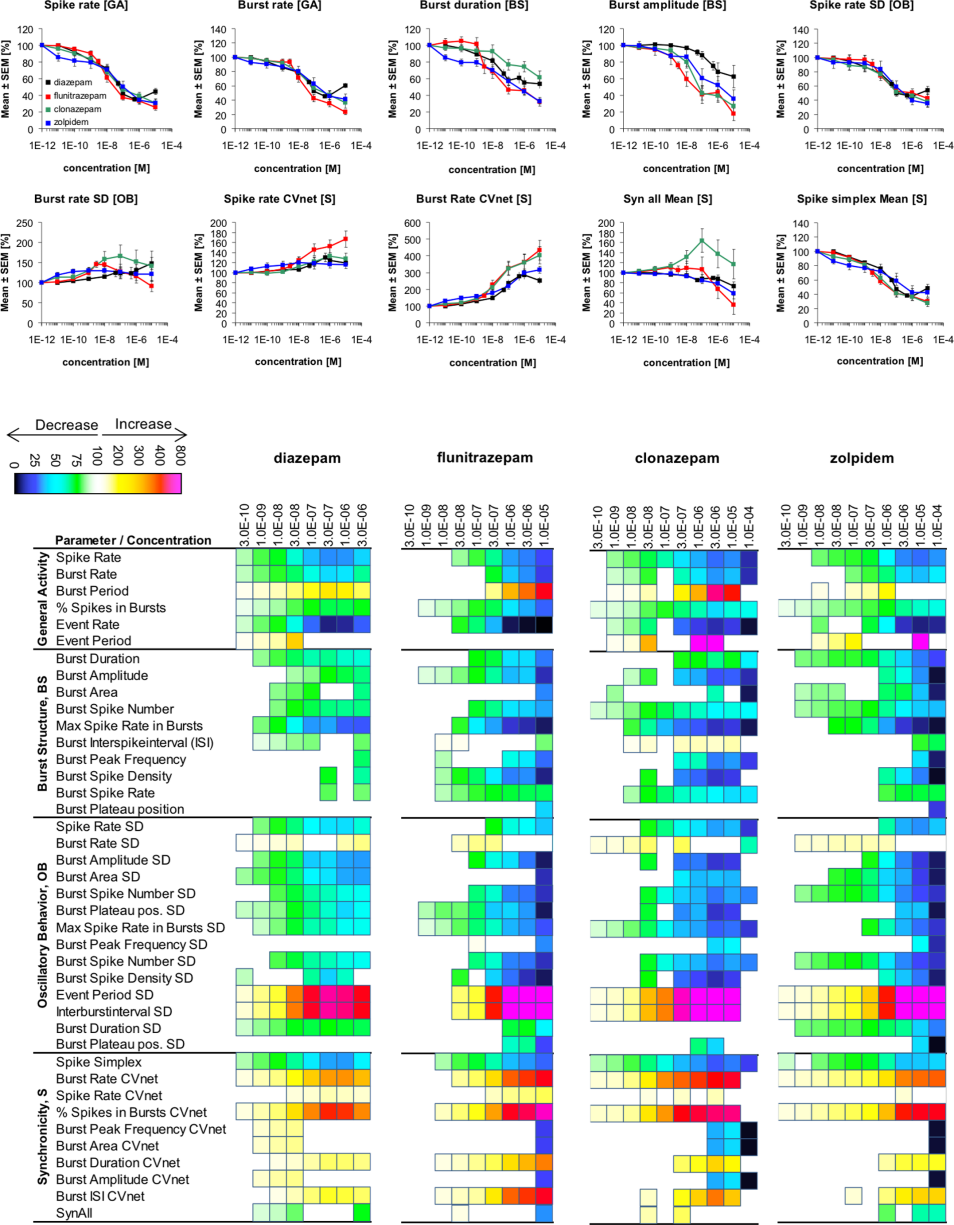
Changes in cortical network activity induced by 8–9 cumulatively increasing concentrations of the αβγ GABA_A_R PAMs diazepam, flunitrazepam, clonazepam and zolpidem. The heat maps present statistically significant changes in 40 activity parameters relative to native activity (no drug, 100%) (Student’s paired t-test, p≤0.05). The concentration-response relationships for the drugs at 10 selected activity parameters are given as mean ± S.E.M. relative to native activity (no drug, 100%).

**Fig 5:**
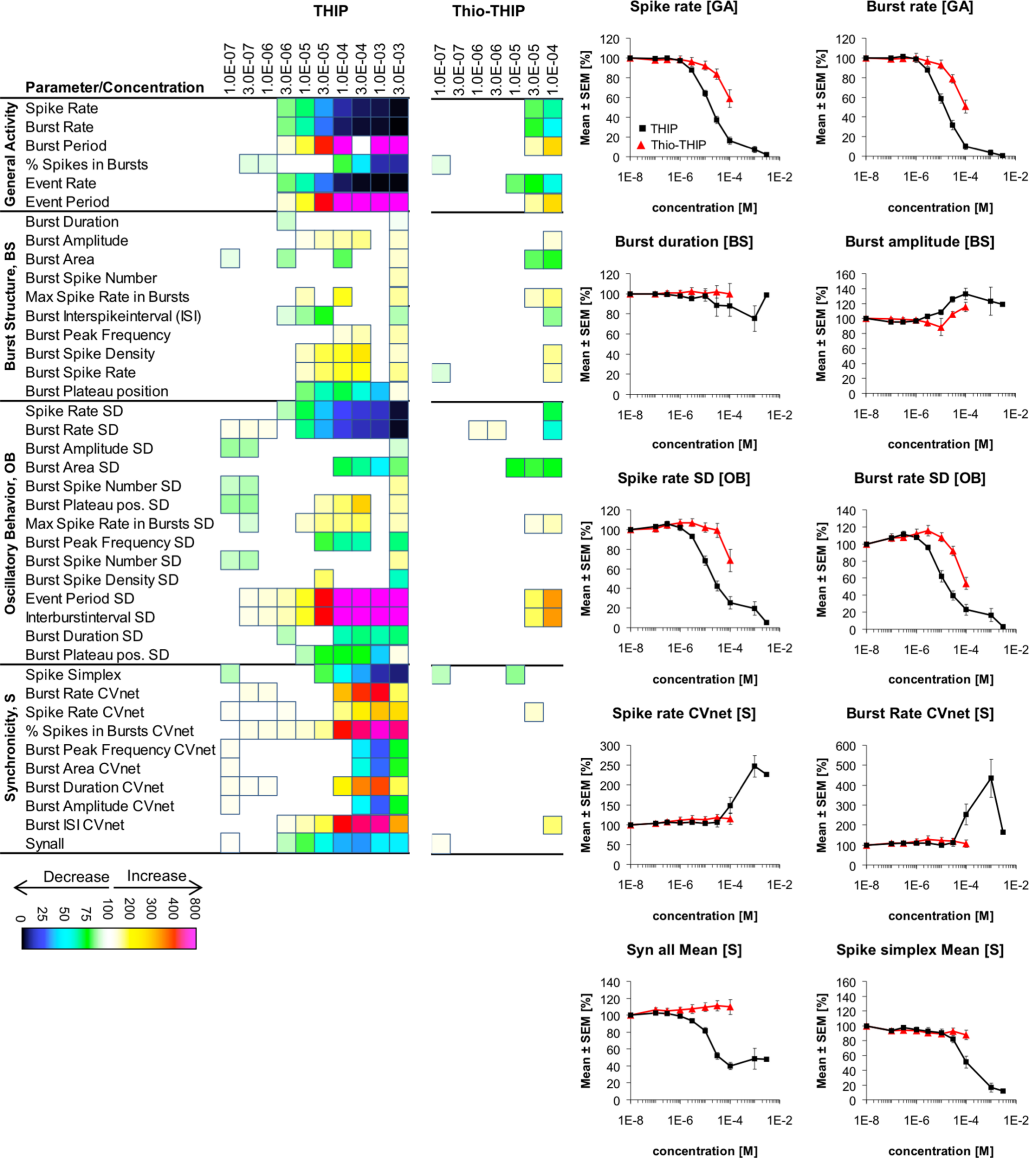
Changes in cortical network activity induced by 7–9 cumulatively increasing concentrations of the functionally selective δ-GABA_A_R agonists THIP and Thio-THIP. The heat maps present statistically significant changes in 40 activity parameters relative to native activity (no drug, 100%) (Student’s paired t-test, p≤0.05). The concentration-response relationships for the drugs at 10 selected activity parameters are given as mean ± S.E.M. relative to native activity (no drug, 100%).

**Fig 6:**
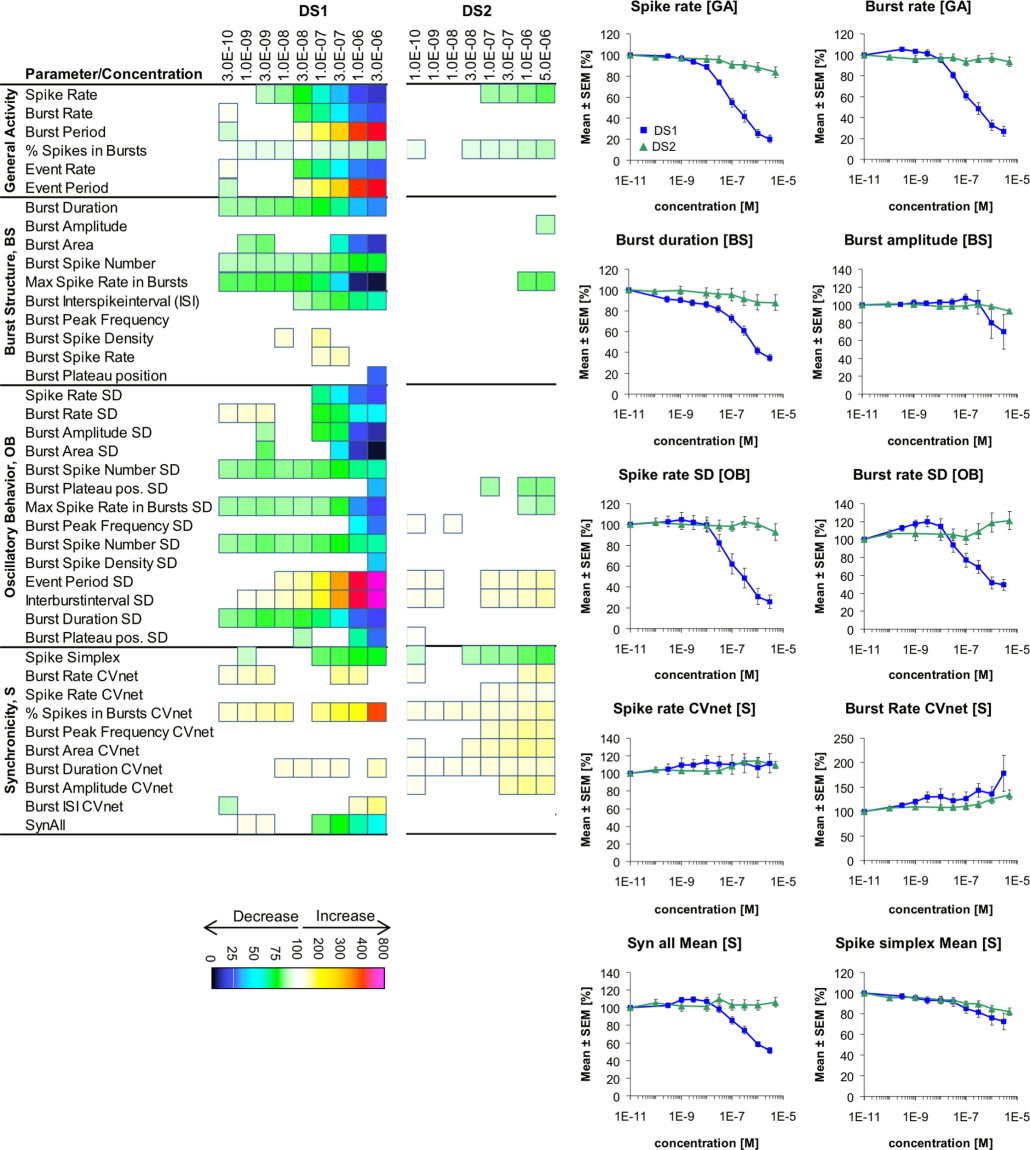
Changes in cortical network activity induced by 8–9 cumulatively increasing concentrations of the δ-GABA_A_R-selective modulators DS1 and DS2. The heat maps present statistically significant changes in 40 activity parameters relative to native activity (no drug, 100%) (Student’s paired t-test, p≤0.05). The concentration-response relationships for the drugs at 10 selected activity parameters are given as mean ± S.E.M. relative to native activity (no drug, 100%).

**Fig 7:**
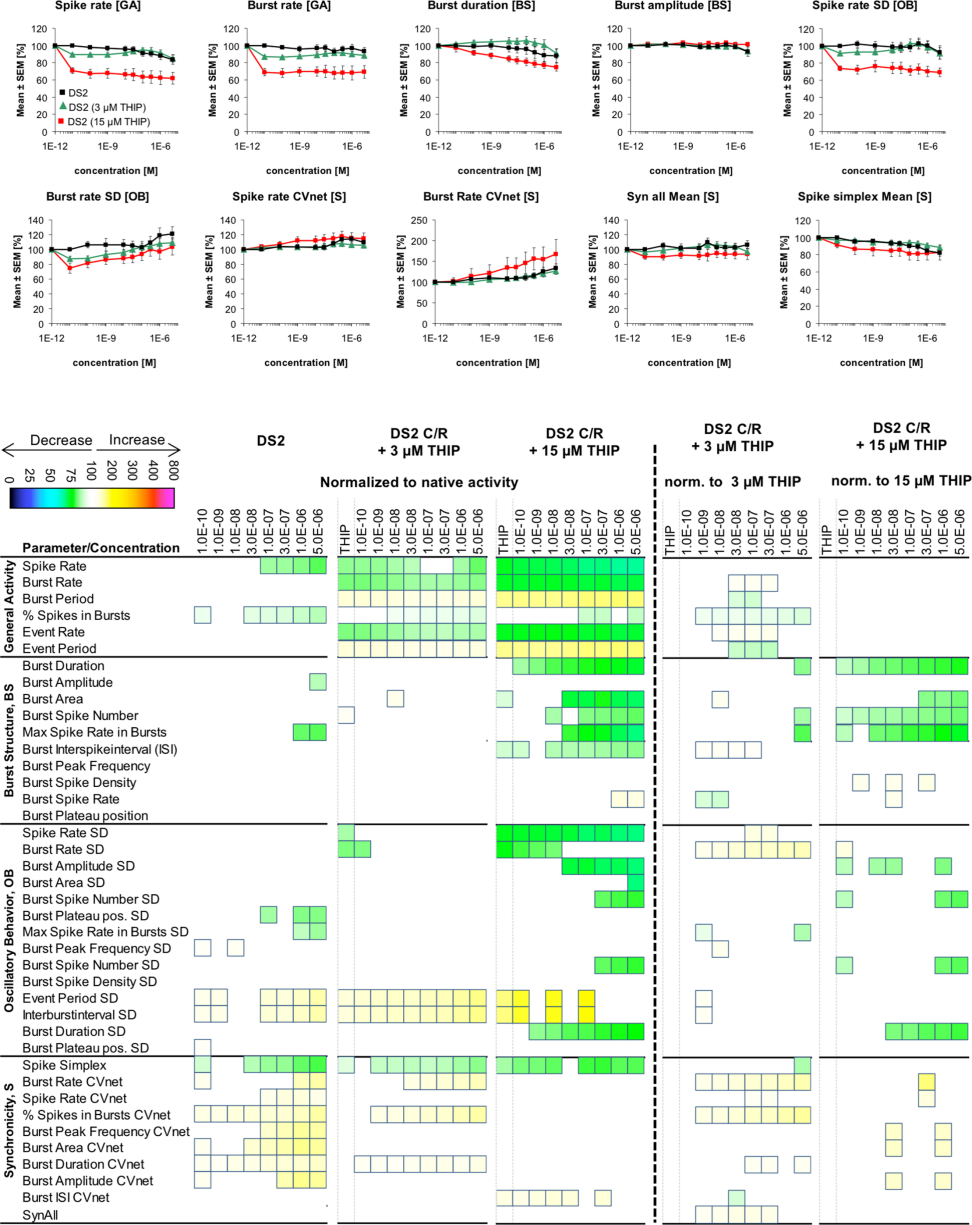
Changes in cortical network activity induced by 8 cumulatively increasing concentrations of DS2 in the absence and in the presence of 3 μM or 15 μM THIP. The heat maps to the left of the vertical hatched line present statistically significant changes in 40 activity parameters relative to native activity (no drug, 100%) (Student’s paired t-test, p≤0.05). The concentration-response relationships for the drug combinations at 10 selected activity parameters are given as mean ± S.E.M. relative to native activity (no drug, 100%). The heat maps to the right of the vertical hatched line present statistically significant changes in the 40 activity parameters between the activity induced by a specific DS2 concentration co-applied with 3 μM or 15 μM THIP relative to the activity induced by THIP on its own (3 μM or 15 μM, respectively) (100%) (Student’s paired t-test, p≤0.05).

**Fig 8:**
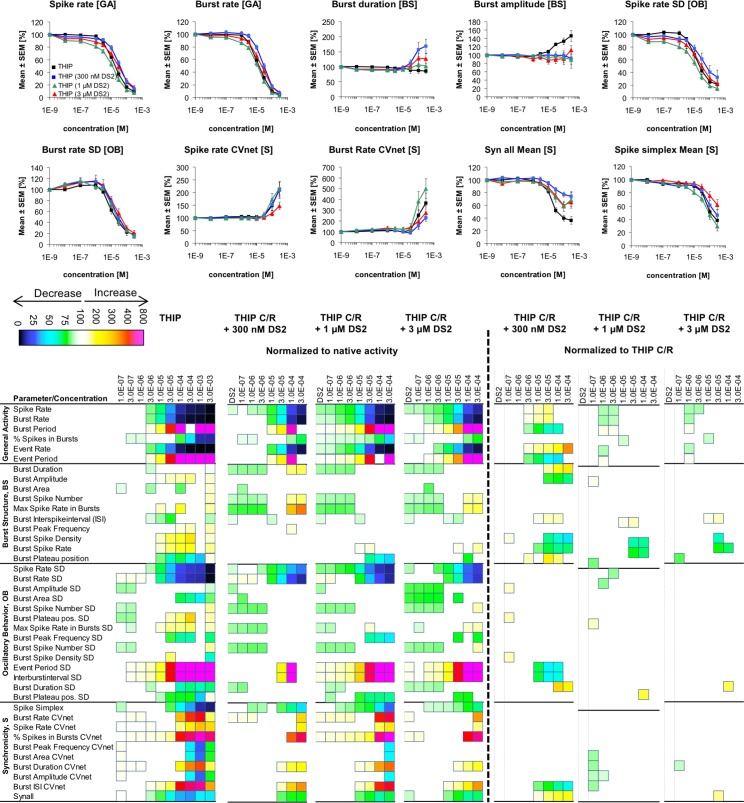
Changes in cortical network activity induced by 7–8 cumulatively increasing concentrations of THIP in the absence and in the presence of 300 nM, 1 μM or 3 μM DS2. The heat maps to the left of the vertical hatched line present statistically significant changes in 40 activity parameters relative to native activity (no drug, 100%) (Student’s paired t-test, p≤0.05). The concentration-response relationships for the drug combinations at 10 selected activity parameters are given as mean ± S.E.M. relative to native activity (no drug, 100%). The heat maps to the right of the vertical hatched line present statistically significant changes in the 40 activity parameters between the activity induced by a specific THIP concentration co-applied with 300 nM, 1 μM or 3 μM DS2 relative to the activity induced by that specific THIP concentration on its own (100%) (Student’s paired t-test, p≤0.05).

**Fig 9:**
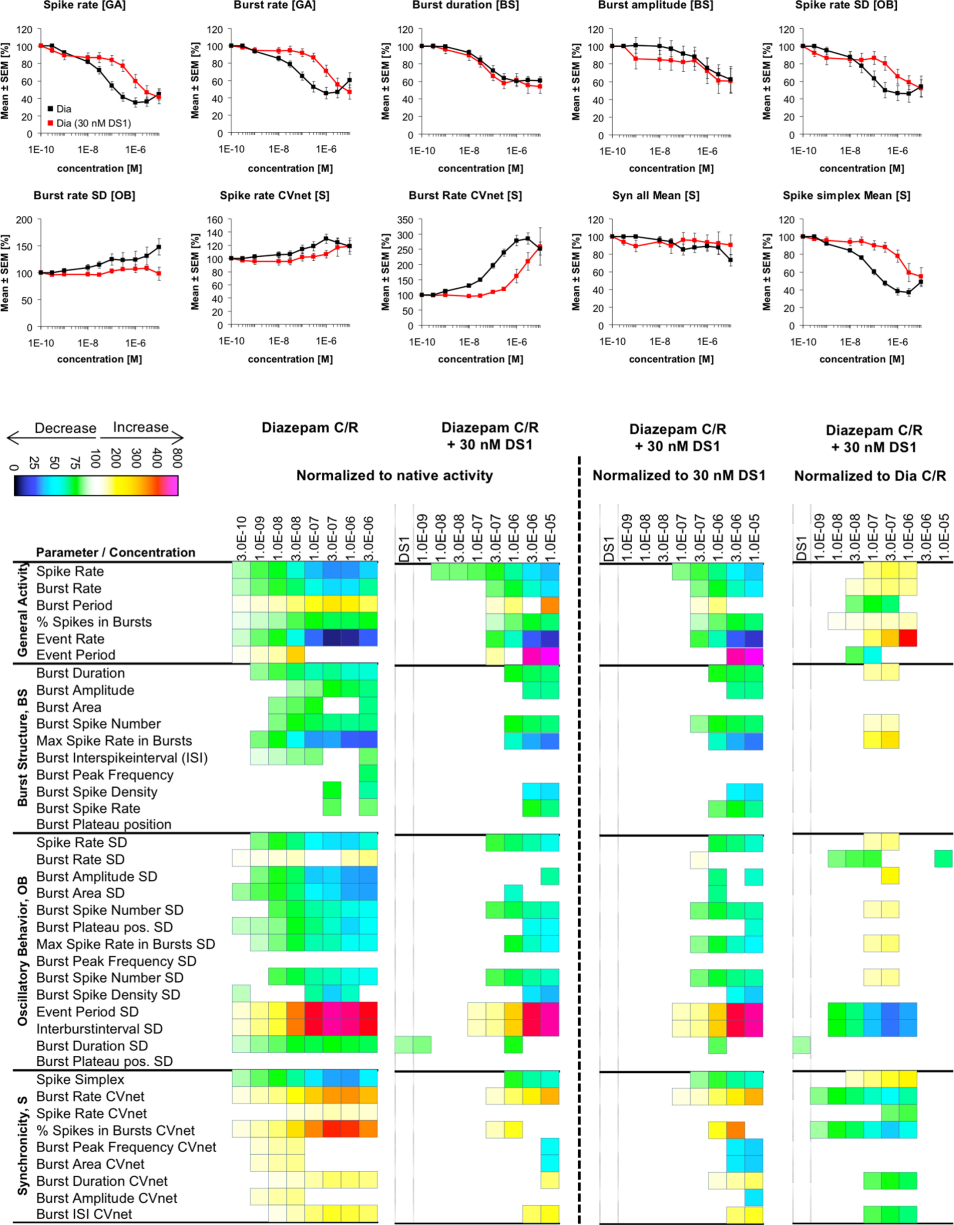
Changes in cortical network activity induced by 8–9 cumulatively increasing concentrations of diazepam in the absence and in the presence of 30 nM DS1. The heat maps to the left of the vertical hatched line present statistically significant changes in 40 activity parameters relative to native activity (no drug, 100%) (Student’s paired t-test, p≤0.05). The concentration-response relationships for the drug combinations at 10 selected activity parameters are given as mean ± S.E.M. relative to native activity (no drug, 100%). The heat maps to the right of the vertical hatched line present statistically significant changes in the 40 activity parameters between the activity induced by a specific diazepam concentration co-applied with 30 nM DS1 relative to the activity induced by 30 nM DS1 on its own (100%) or relative to the activity induced by that specific diazepam concentration on its own (100%) (Student’s paired t-test, p≤0.05).

**Fig 10:**
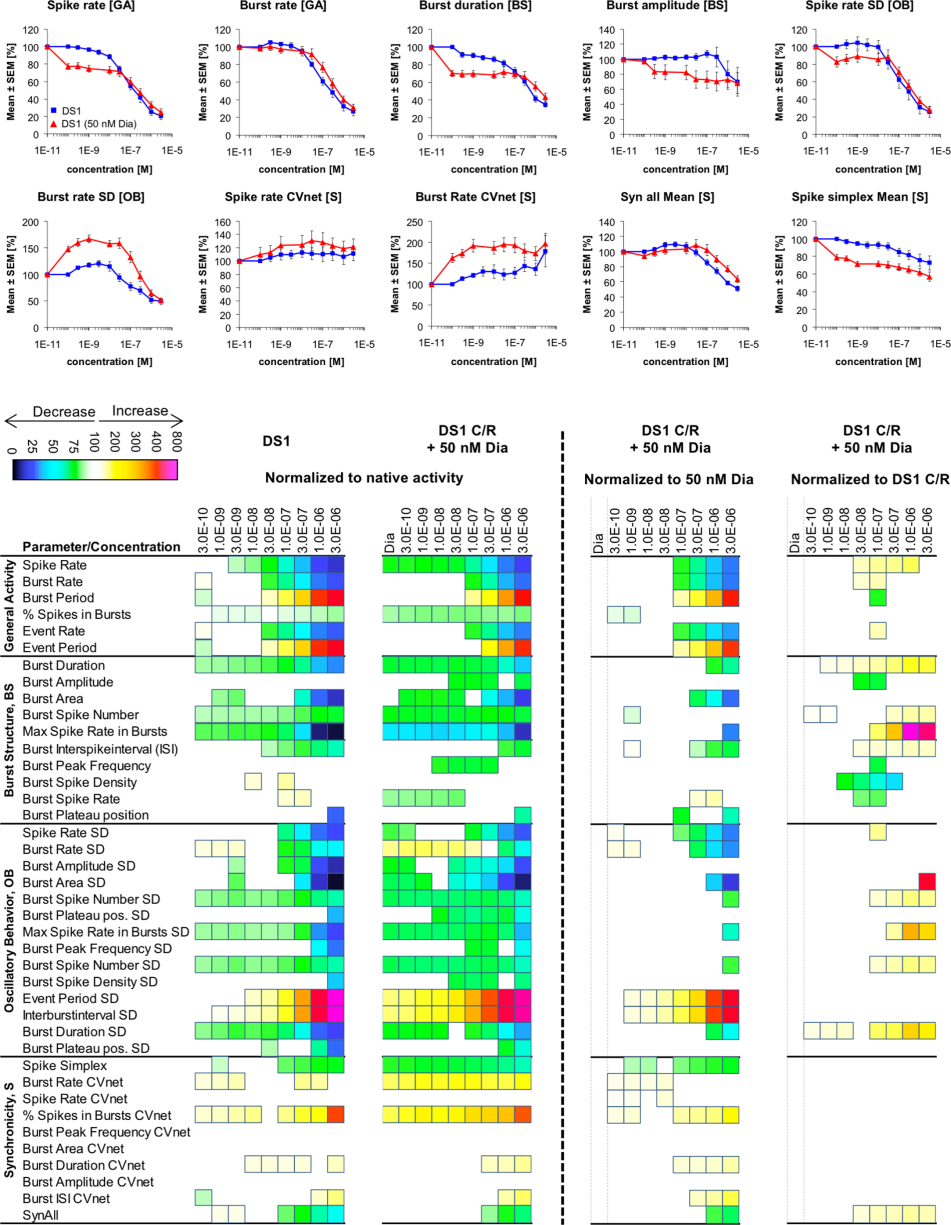
Changes in cortical network activity induced by 8–9 cumulatively increasing concentrations of DS1 in the absence and in the presence of 50 nM diazepam. The heat maps to the left of the vertical hatched line present statistically significant changes in 40 activity parameters relative to native activity (no drug, 100%) (Student’s paired t-test, p≤0.05). The concentration-response relationships for the drug combinations at 10 selected activity parameters are given as mean ± S.E.M. relative to native activity (no drug, 100%). The heat maps to the right of the vertical hatched line present statistically significant changes in the 60 activity parameters between the activity induced by a specific DS1 concentration co-applied with 50 nM diazepam relative to the activity induced by 50 nM diazepam on its own (100%) or relative to the activity induced by that specific DS1 concentration on its own (100%) (Student’s paired t-test, p≤0.05).

The principles of the MEA recordings, the experimental set-up, the raw data obtained from the recordings, and the subsequent multi-parametric data analysis performed in this study are illustrated in Fig 2A. The 204 activity-describing parameters calculated based on the spike trains from the multichannel recordings are divided into four categories (Fig 2A). *“General Activity”* parameters are global network activity descriptors (e.g. spike rate, burst rate, % of spikes in bursts and burst period), whereas *“Burst Structure”* parameters describe the internal structure of spikes within a high-frequency spiking phase (e.g. spike frequency in bursts, spike rate in bursts, spike density) as well as the overall burst structure (e.g. the duration, area, plateau of the burst). *“Oscillatory Behavior”* parameters are the standard deviations associated with main *General Activity* and *Burst Structure* parameters, and these parameters are thus descriptors of the regularity of bursting events within experimental episodes, with higher values indicating less regular general activity or less regular burst structure. Finally, the *“Synchronicity”* parameters include those representing the coefficient of variation over the network. Thus, the *Synchronicity* CVnet values are reflecting the level of synchronization amongst the neurons, i.e. the degree of variation for the respective *General Activity* or *Burst Structure* parameter between individual units in a network. The lower the CVnet, the higher the synchronicity among neurons and vice versa. The 40 activity parameters shown in heat maps in Figs 3–10 represent a wide view on concentration-dependent effects of agents and thus the phenotypic fingerprint which is unique to every compound but similar between compounds with same modes of action. Full names and definitions of the 40 parameters are given in S1 Table.

Under optimal culture conditions the MEA cultures display stable activity levels for several hours with only a few parameters exhibiting changed properties over time (Fig 2B). Moreover, DMSO mediates either no significant effects or negligible effects on all activity parameters when applied at assay concentrations up to 0.1%. This stability allows measurements of the effects induced by cumulatively increasing concentrations of a drug on the same network in recordings over a time period of 8–9 h and the subsequent normalization the all activity parameter data for a specific drug concentration to the intrinsic native activity recorded (control). In conclusion, robust data describes the complete concentration-response relationships for all activity parameters for the drugs.

Because of the holistic nature of the information extractable from MEA recordings on neuronal networks, there are aspects of GABA_A_R signaling and its contributions to overall GABAergic transmission that may be addressed in slice and patch-clamp recordings at neurons but not in these recordings. In the present study, application of cumulatively increasing concentrations of the different GABAergic agonists and PAMs did not lead to visible desensitization of the induced changes in the activity parameters over time. That is not to say that exposure of the networks to these drugs (especially at high concentrations) does not induce desensitization of individual GABA_A_Rs in the neurons. However, this ongoing desensitization (and subsequent resensitization) of individual receptors in the neurons either does not manifest itself significantly in the measured read-outs in the recordings or this desensitization may actually be imbedded in the observed changes in the various activity parameters induced by the drugs. Furthermore, it is also not possible to differentiate between the respective contributions of presynaptic and postsynaptic GABA_A_Rs to the total effects at network activity induced by a specific drug in the MEA recordings. On the other hand, provided that activity patterns in the pre-frontal cortex cultures used for the MEA recordings to a reasonable degree are reflective of cortical activity patterns *in vivo*, we propose that the drug-induced network activity changes in this system can offer interesting information about other aspects of GABA_A_R-mediated functions in an *in vivo* setting.

### Muscimol and baclofen

Initially the effects on cortical network activity produced by selective activation of GABA_A_Rs or GABA_B_Rs were studied by applications of muscimol and baclofen, respectively. Muscimol is a pan-GABA_A_R agonist displaying full agonism and EC_50_ values in the high nanomolar/low micromolar range at various receptor subtypes expressed in oocytes or mammalian cells [40–42], and baclofen is a selective full GABA_B_R agonist exhibiting low micromolar EC_50_ values at the recombinant GABA_B_Rs [43–45]. In the MEA recordings, muscimol and baclofen were applied at the networks at concentrations up to 3 μM and 10 μM, respectively, and activity changes induced by the two drugs must thus be assumed to arise from substantial (if not maximal) degrees of activation of GABA_A_Rs and GABA_B_Rs, respectively. Importantly, the concentration-response relationships exhibited by muscimol (EC_50_ ~100 nM) and baclofen (EC_50_ ~1 μM) at major *General Activity* parameters such as *“Spike Rate”* and *“Burst Rate”* were in concordance with the reported potencies for the two agonists at their respective recombinant receptors. Muscimol and baclofen mediated very similar changes in the *General Activity* parameters, with both drugs inducing overall suppression of network activity in the form of decreased spike and burst rates and increased burst periods and inter-burst intervals (Fig 3). In contrast, the two drugs modulated several *Burst Structure* parameters differently, with muscimol decreasing burst durations and amplitudes and baclofen inducing the opposite effects (Fig 3). Moreover, whereas GABA_A_R activation mainly decreased the variation of *General Activity* and *Burst Structure* parameters between different bursts, GABA_B_R activation had more subtle impact on this variation and in some cases even increased it (*Oscillatory Behavior* parameters, Fig 3). Thus, the changes in neuronal network activity resulting from stimulation of GABA_A_R and GABA_B_R signalling were phenotypically distinct. In the subsequent experiments we probed the contributions of different receptor populations to the GABA_A_R-mediated effects.

### Positive allosteric modulators (PAMs) of αβγ GABA_A_Rs

To assess the network effects arising from selective augmentation of synaptic αβγ GABA_A_R signalling, the benzodiazepines diazepam, flunitrazepam and clonazepam and the benzodiazepine-site PAM zolpidem were characterized at the neuron cultures. All these four PAMs act through the high-affinity benzodiazepine binding sites formed at the extracellular α^(+)^/γ^(–)^ subunit interface in α_1,2,3,5_βγ GABA_A_Rs, and in the concentration ranges used in the MEA recordings the modulators will act exclusively at these receptors without affecting other subtypes. Diazepam, flunitrazepam and clonazepam are fairly non-selective PAMs, and the network effects mediated by these modulators must thus be assumed to arise from modulation of all α_1,2,3,5_βγ receptors [46–49]. In contrast, zolpidem is α_1_-preferring PAM exhibiting EC_50_ values of ~100 nM at α_1_βγ receptors, ~10-fold higher EC_50_ values at α_2_βγ and α_3_βγ receptors and negligible activity at α_5_βγ receptors in heterologous expression systems [48, 50–52]. Thus, the network effects mediated by low concentrations of zolpidem must predominantly be ascribed to α_1_βγ receptors, whereas α_2_βγ and α_3_βγ subtypes are likely to contribute the effects observed at higher concentrations of the modulator.

All in all, diazepam, flunitrazepam, clonazepam and zolpidem induced similar effects at the neuronal networks in the MEA recordings (Fig 4). However, the degrees of modulation exerted by the four PAMs differed somewhat at some of the activity parameters, most notably flunitrazepam exerted significant effects at more *Burst Structure* parameters than the other PAMs. The changes in the various activity parameters brought on by the four PAMs were, with a few exceptions, in the same qualitative directions as those induced by muscimol. Interestingly, however, sub-saturating concentrations of muscimol induced considerably more pronounced effects on major *General Activity* parameters such as *“Spike Rate”* and *“Burst Rate”* than saturating concentrations of the PAMs (Figs 3 and 4). This difference could be rooted in this pan-GABA_A_R agonist targeting a broader spectrum of GABA_A_R subtypes than the benzodiazepine-site PAMs. Alternatively, since the PAMs are dependent on concomitant agonist binding to the receptors to mediate their effects, the maximal degrees of modulation induced by them in these recordings could also be limited by the endogenous concentration of GABA in the assay.

In concordance with their somewhat higher potencies as PAMs at recombinant α_1,2,3,5_βγ receptors, flunitrazepam and clonazepam were observed to be slightly more potent than diazepam at most *General Activity* and *Burst Structure* parameters. Interestingly, the degrees of modulation induced by 100 nM zolpidem and 100 nM diazepam at the various activity parameters were largely comparable (Fig 4). Since diazepam and zolpidem are essentially equipotent PAMs at α_1_βγ receptors expressed in *Xenopus* oocytes (EC_50_ values ~100 nM) [47, 48, 50, 51], the two modulators must be assumed to exert comparable degrees of α_1_βγ receptor modulation at this concentration, whereas diazepam (100 nM) in contrast to zolpidem (100 nM) also will mediate substantial modulation of α_2,3,5_βγ receptors. Thus, the comparable effects of the two modulators is likely to be a reflection of the fact that α_1_βγ receptors (including α_1_α_x_βγ receptors comprising an α_1_^(+)^/γ^(–)^ subunit interface) are the major synaptic GABA_A_R subtypes in cortical neurons [2].

### THIP and Thio-THIP

The orthosteric GABA_A_R ligands THIP and Thio-THIP both exhibit functional selectivity for δ-GABA_A_Rs over other receptor subtypes. While THIP is an agonist at a wide range of GABA_A_Rs, it exhibits substantially higher maximal responses (relative to GABA) at δ-GABA_A_Rs than at αβγ receptors [42, 53–55]. More importantly, THIP is also a more potent agonist at α_4_βδ receptors than at αβγ and αβ receptors, and this selectivity for δ-GABA_A_Rs within a certain concentration range has made THIP the prototypic pharmacological tool for these receptors [42, 53–56]. The close structurally related analog Thio-THIP is a functionally subtype-selective α_4_βδ GABA_A_R ligand, as it acts as a partial agonist at α_4_β_1_δ and α_4_β_3_δ receptors and exhibits negligible agonist activity at α_4_β_2_δ and αβγ GABA_A_Rs [57].

Both THIP and Thio-THIP induced significant changes in cortical network activity in a concentration-dependent manner (Fig 5). THIP exhibited EC_50_ values in the 10–30 μM range at the majority of the *General Activity* parameters, and applications of 3 μM or 10 μM THIP produced significant effects at most of the activity parameters (Fig 5). In electrophysiological recordings at recombinant GABA_A_Rs expressed in *Xenopus* oocytes or mammalian cells, THIP has been found to induce α_4_βδ receptor signaling without concomittant activation of αβγ receptors within a concentration range spanning from high-nanomolar concentrations to approximately 10 μM [42, 54, 55, 57], which indicate that the significant network effects induced by THIP at 3 and 10 μM concentrations could be mediated predominantly through δ-GABA_A_Rs. Conversely, in slice recordings high-nanomolar concentrations of THIP have been shown to induce robust tonic currents in neurons expressing α_4_βδ GABA_A_Rs as major extrasynaptic receptors [57–60], suggesting that significant network effects perhaps could have been expected to arise from lower THIP concentrations than 3/10 μM. Thus, if one were to go by the ability of THIP to induce tonic currents at high-nanomolar concentrations the fact that concentrations of 3 μM and higher were required to induce signficant network effects could be interpreted either as lack of expression of δ-GABA_A_Rs in the neurons or as a reflection of negligible contributions of δ-GABA_A_R signaling to network activity. On the other hand, even though the low degree of δ-GABA_A_R occupancy and activation seen at high-nanomolar concentrations of THIP is able to tonic currents there does not necessarily have to be a 1:1 correlation between its effects at these concentrations and the THIP concentrations capable of giving rise to significant changes in the activity parameters in the MEA recordings. In conclusion, although the observation that THIP first induces significant networks effects at 3/10 μM concentrations where the agonist is known to begin to activate αβγ and other GABA_A_Rs as well could be taken as an indication that its network effects could arise from these receptors rather than from δ-GABA_A_Rs, it could also mean that the δ-GABA_A_R-mediated effects on network activity requires a higher degree of receptor occupancy by THIP. In the latter case, the networks effects induced by THIP (3/10 μM) could be mediated through both αβγ and δ-GABA_A_Rs.

Whereas THIP could be tested at the networks at concentrations up to 3 mM, application of Thio-THIP at concentrations of 300 μM and higher resulted in neurotoxic effects. Thus, Thio-THIP was only tested at concentrations up to 100 μM, where these effects were not observed. Within this concentration-range Thio-THIP mediated changes in substantially fewer network activity parameters than THIP, and significant effects were mostly seen at concentrations of 30 μM and 100 μM (Fig 5). In view of its negligible activity at recombinant αβγ GABA_A_Rs at concentrations up to 100 μM, the effects of Thio-THIP on network activity most likely arise from its agonism at δ-GABA_A_Rs, and the fact that significant effects typically only arises from compound concentrations of 30 μM or 100 μM seems to concord with its potency as a α_4_βδ receptor agonist. Moreover, judging from its functional selectivity profile at the three recombinant α_4_βδ subtypes these effects are likely to be mediated through α_4_β_1_δ and/or α_4_β_3_δ receptors [57].

### DS1 and DS2

Next the close structurally related allosteric ligands DS1 and DS2 were tested at the cortical networks. DS1 and DS2 have been reported to be selective modulators of δ-GABA_A_R (in particular α_4_βδ and α_6_βδ) over α_1,2,3,5_β_2_γ_2_ and α_4_β_3_ receptors expressed in oocytes and mammalian cells [61, 62]. DS1 exhibits pronounced intrinsic activity and thus acts both as an allosteric agonist and a PAM (ago-PAM) at recombinant α_4_β_3_δ receptors, whereas DS2 has been reported to be a pure PAM at these receptors [61, 62]. Importantly, however, both compounds modulate αβγ receptors in the same concentration range as δ-GABA_A_Rs, and the proposed δ-GABA_A_R selectivity of these compounds are thus rooted in the dramatically higher efficacies exhibited by them as modulators of these receptors than at the αβγ receptors [54].

In the MEA recordings DS1 and DS2 were both applied in concentration ranges that cover most of their effective concentration-response relationships at recombinant α_4_β_3_δ GABA_A_Rs [54, 61, 62]. Interestingly, DS2 either mediated very subtle effects or no significant effects at the various activity parameters at concentrations up to 5 μM (the highest concentration tested due to restrictions in terms of the maximum assay concentration of DMSO), whereas DS1 induced pronounced effects in a concentration-dependent manner at the majority of parameters in all four classifications (Fig 6). Importantly, significant effects were observed from applications of even low nanomolar DS1 concentrations, and the ago-PAM exhibited EC_50_ values in the 100–300 nM range at several of the major *General Activity* and *Burst Structure* parameters, which is in agreement with its reported potency at recombinant α_4_βδ receptors [54, 61]. The DS1-induced changes in the activity parameters were all in the same directions as those mediated by THIP (3/10 μM) and by Thio-THIP (Fig 5), and the changes were also phenotypically similar to those produced by the benzodiazepine-site PAMs (Fig 4). Assuming that the network effects mediated by THIP (3/10 μM) and Thio-THIP predominantly can be ascribed to δ-GABA_A_R activation, the substantially more pronounced changes in the activity parameters mediated by DS1 could be ascribed to the higher efficacy of this ago-PAM at δ-GABA_A_Rs than THIP (3/10 μM) and the partial agonist Thio-THIP [42, 54, 55, 57, 61].

We found the dramatically different efficacies of DS1 and DS2 in the MEA recordings interesting. The DS2 sample used in the MEA recordings was found to exhibit robust PAM activity at recombinant α_4_β_3_δ GABA_A_Rs expressed in *Xenopus* oocytes, and thus the negligible effects of the PAM at cortical network activity could not be ascribed to lack of δ-GABA_A_R activity in this specific sample. Moreover, as outlined above the effects mediated by DS1, Thio-THIP and, to some extent, THIP suggest that augmentation of δ-GABA_A_R signaling is impact network activity. Thus, we speculated that the negligible effects mediated by DS2 could be rooted in GABA concentrations surrounding extrasynaptic δ-GABA_A_Rs in the neurons being too low to enable the PAM to mediate its effects. To investigate this possibility, we studied the network effects induced by DS2 in the presence of a trace concentration of THIP. Co-application of DS2 (0.1 nM-5 μM) with 3 μM THIP produced very subtle changes in the activity parameters, both when compared to the activity recorded in native cells and to the activity arising from the initial application of 3 μM THIP (Fig 7). Even when DS2 (0.1 nM-5 μM) was co-applied with 15 μM THIP, a concentration of the agonist that on its own induced significant changes in several activity parameters and thus must be assumed to exert robust δ-GABA_A_R activation, the effects induced by the PAM were subtle albeit more substantial than those induced in the presence of 3 μM THIP (Fig 7). Interestingly, DS2 predominantly augmented the 15 μM THIP-mediated effects at *Burst Structure* parameters, whereas the *General Activity* parameters were largely unaffected by the presence of the modulator (Fig 7).

In reverse experiments, the concentration-response relationships for THIP at the network activity parameters were determined in the presence of 300 nM, 1 μM or 3 μM DS2, three concentrations that cover most of the effective concentration range of DS2 (~EC_30_-EC_90_) at recombinant α_4_β_3_δ GABA_A_Rs [54, 61, 62]. The presence of DS2 during these recordings only impacted the concentration-response relationships exhibited by THIP slightly, the most substantial modulation being observed at lowest PAM concentration (Fig 8). The presence of 300 nM DS2 in the assay right-shifted the concentration-response relationships displayed by THIP or reduced its efficacy at several *General Activity* and *Burst Structure* parameters (Fig 8). Albeit this modulation was subtle this DS2 concentration thus seemed to counteract the network effects arising from THIP-mediated δ-GABA_A_R modulation, whereas the presence of higher concentrations of the modulator (1 μM and 3 μM) generally were without effect (Fig 8).

### Diazepam and DS1 combinations

Having studied the changes in network activity mediated by selective modulators of αβγ GABA_A_Rs and δ-GABA_A_Rs, we next set out to investigate the putative functional interplay between the two receptor classes in the neurons, i.e. to which extent concomittant δ-GABA_A_R signalling would impact the network changes mediated by αβγ GABA_A_Rs and vice versa. DS1 and diazepam were applied as selective mediators of signalling through these two receptor classes in the recordings, where the concentration-response relationships for one modulator were determined at the networks in the concomitant presence of a fixed low concentration of the other. Concentrations of 30 nM DS1 and 50 nM diazepam were used for the recordings, since these concentrations of the respective drugs produced small but significant changes in the majority of activity parameters in the original recordings (Figs 4 and 6).

The network activity changes induced by diazepam (1 nM-10 μM) in the presence of 30 nM DS1 were all characterized by the same directions as those induced by the benzodiazepine on its own (Fig 9). In these recordings, application of 30 nM DS1 on its own induced more modest effects on the activity parameters than expected based on its concentration-response relationships in the original DS1 recordings (Fig 6). Nevertheless, when co-applied with this trace concentration of DS1 diazepam was strikingly less efficacious at the networks than when applied on its own. In fact, the concentration-response relationships exhibited by the “diazepam (30 nM DS1)” combination at numerous activity parameters (including major *General Activity* and *Burst Structure* parameters such as “*Spike rate*”, “*Burst rate*”, “*Burst duration*” and “*Burst amplitude*”) were dramatically right-shifted compared to those evoked by diazepam on its own, in some cases up to ~30-fold (Fig 9). Thus, the concomittant presence of this low DS1 concentration appeared to decrease the sensitivity of the neurons to the diazepam-mediated αβγ GABA_A_R signalling.

In another experiment the roles of the two modulators were reversed, and DS1 (0.1 nM-3 μM) was co-applied with 50 nM diazepam at the networks (Fig 10). In agreement with the original diazepam recordings (Fig 4), application of 50 nM diazepam mediated significant effects on the majority of activity parameters, inducing responses constituting up to ~30% of the responses induced by saturating diazepam concentrations (Fig 10). The network activity changes arising from DS1 in the presence of 50 nM diazepam were all characterized by the same qualitatively directions as those induced by DS1 on its own. Moreover, when the isolated effects mediated by 50 nM diazepam on the networks were taken into account, the concentration-response relationships exhibited by the “DS1 (50 nM diazepam)” combination at the activity parameters either did not differ signifiantly or were only slightly different (mostly right-shifted) compared to those displayed by DS1 on its own (Fig 10). Thus, in contrast to the observation made in the reverse experiment, a moderate level of stimulation of αβγ GABA_A_Rs thus did not seem to affect the network effects mediated by DS1 in this experimental set-up substantially.

## Discussion

In the present study the effects of various pharmacological tools characterized by different modulatory properties and selectivity profiles at GABA_A_Rs on cortical neuronal network activity were characterized investigated by use of MEA recordings and multiparametric data analysis. We report that selective augmentation of GABA_A_R and GABA_B_R signaling by the pan-agonists muscimol and baclofen, respective, mediate phenotypically distinct changes in network activity, and that the networks effects induced by wide range of GABA_A_R modulators with different subtype-selectivity profiles largely were in the same qualitative directions, albeit with some interesting exceptions. Thus, benzodiazepine-site PAMs selective for αβγ GABA_A_Rs as well as the reported δ-GABA_A_R-selective agents DS1 and Thio-THIP induced robust changes in a broad range of activity parameters. Interestingly, the efficacious modulation of network activity mediated by the ago-PAM DS1 was contrasted by the negligible effects exhibited by its close structural analog DS2, another reported δ-GABA_A_R-selective PAM, at the neurons. Finally, in studies of the putative functional interplay between αβγ GABA_A_Rs and δ-GABA_A_R, co-administration of diazepam with a low but effective concentration of DS1 was found to decrease the apparent potency of the bezodiazepine at numerous activity parameters, whereas low levels of concomittant αβγ GABA_A_R stimulation had little impact on the DS1-induced network effects.

Interpretations made in any study probing native GABA_A_R signaling with pharmacological tools are obviously reliant on the reported functional properties of these at recombinant receptors being accurate and as detailed as possible. Furthermore, although functionalities of ligands determined at recombinant GABA_A_Rs usually are assumed to be mirrored at the native receptors, this may not always be the case. This is particular true for the α_4_βδ GABA_A_Rs that assemble into different functional receptor stoichiometries and arrangements in heterologous expression systems that may not all exist *in vivo* [63–67]. Thus, even though the αβγ GABA_A_R selectivity of the benzodiazepine-site PAMs [46–52], the efficacy-based functional selectivity of DS1 and DS2 as δ-GABA_A_R modulators [54, 61, 62], and the functional δ-GABA_A_R selectivity of THIP and Thio-THIP [42, 56, 57] are well-founded in the literature, such considerations are also valid for this study. It is also important to stress that while α_1,2,3_βγ GABA_A_Rs and δ-GABA_A_Rs (mainly α_4_βδ) are the predominant synaptic and extrasynaptic receptors in cortical neurons, other subtypes are likely to be expressed at both sites in these neurons and to contribute to phasic and tonic inhibition as well, analogously to what has been demonstrated in other brain regions [11, 13, 68–70]. The reported expression of extrasynaptic α_5_βγ receptors in layer 5 cortical neurons [71] is particular relevant, since the networks effects induced by diazepam, flunitrazepam and clonazepam thus potentially could comprise a component arising from activation of these receptors. As outlined in *Results*, however, the similarity of the networks effects mediated by the benzodiazepines to those of zolpidem, a PAM with negligible α_5_βγ activity, strongly suggests that the effects predominantly are attributable to activation of α_1,2,3_βγ receptors (Fig 4). Thus, while keeping the heterogeneity of native GABA_A_Rs and the other aspects outlined above in mind, we propose that the GABA_A_R ligands applied in this study relatively specifically delineate the networks effects arising from augmentation of the signalling of αβγ GABA_A_Rs and δ-GABA_A_Rs.

Another general concern in studies of GABA_A_R-mediated effects in neuronal cultures is obviously to which extent the relative distribution and the expression levels of various receptor subtypes in the cultures mirror those in native tissues and to which degree effects observed for various pharmacology tools here can be extrapolated to the *in vivo* situtation. As evidenced by the substantial expression levels detected for the α_1_, α_3_ and β_1_-β_3_ subunits in neuronal culture lysates and by the robust network activity changes mediated by four benzodiazepine-site PAMs in concentration ranges in good agreement with their modulatory potencies at recombinant αβγ GABA_A_Rs (Figs 1 and 4), there is little doubt that αβγ GABA_A_Rs are expressed in the primary neurons and are of major importance for the level of spontaneous activity in these networks. However, when it comes the δ-GABA_A_Rs, the data presented in this study is admittedly more ambiguous and open to interpretation. Even through the significantly lower expression levels of α_4_ and δ subunits compared to β_1_-β_3_ in the primary neurons are in concordance with the relative expression of these subunits in postnatal mouse frontal cortex (Fig 1E), it is reasonable to question whether δ-GABA_A_Rs expressed at such low levels would be able to exert substantial effects on network activity. Unfortunately, the network effects mediated by the four drugs applied in this study to elucidate the importance of these δ-GABA_A_Rs are also quite ambiguous. The fact that DS1 and Thio-THIP mediate robust modulation of the majority of activity parameters and at these exhibit concentration-response relationships that concord with their respective potencies at recombinant δ-GABA_A_Rs seem to support the notion of these receptors being important for spontaneous activity in the networks (Figs 5 and 6). As outlined in detail in the *Results* section, the concentration-response relationship exhibited by THIP in its modulation of network activity can both be argued to substantiate and to disprove an involvement of δ-GABA_A_Rs, and as such the data for this drug does not offer clarity on the issue. Finally, the negligible modulation of network activity mediated by the δ-selective PAM DS2, both when applied on its own and with THIP, conversely suggests that the contributions of δ-GABA_A_Rs to network activity changes could be minute (Figs 6–8). However, in contrast to its potent and highly efficacious modulation of recombinant α_4_βδ GABA_A_Rs, DS2 is not a particular potent inducer of tonic inhibition through native α_4_βδ receptors in murine thalamic ventrobasal neurons, where it only elicits significant tonic currents at a concentration of 3 μM [61, 62]. Thus, the fact that DS2 in this study only was tested at concentrations up to 5 μM could perhaps explain its modest effects on network activity. In conclusion, we favor an interpretation of these data where the observed effects of DS1, Thio-THIP and, to some extent, THIP (3/10 μM) on network activity are mediated predominantly through δ-GABA_A_Rs. Although we can not completely exclude the possibility that the low expression levels of δ-GABA_A_Rs in the neurons could mean that these receptors do not contribute significantly to the observed network activity changes, any alternative explanation would require both DS1 and Thio-THIP to mediate their robust network effects through other targets than δ-GABA_A_Rs, which we find a more unlikely scenario.

The dramatically different modulation of network activity mediated by the close structural analogs DS1 and DS2 in the MEA recordings is truly striking. As mention above, we propose that the DS1-mediated effects are arising from δ-GABA_A_R modulation, and the effective concentration ranges exhibited by DS1 at the various activity parameters are certainly in good agreement with the reported potencies for it as an ago-PAM at recombinant α_4_βδ GABA_A_Rs (Fig 6) [54, 61]. To our knowledge, there are no published studies of the modulation exerted by DS1 at native δ-GABA_A_Rs, and thus it remains to be seen whether DS1 analogously to DS2 displays different modulatory potency in its induction of tonic currents in slice recordings than at recombinant δ-GABA_A_Rs. However, considering that DS1 is a ~10-fold more potent PAM at recombinant α_4_βδ GABA_A_Rs than DS2 and, in contrast to DS2, also exhibits pronounced intrinsic agonist activity at the receptors [54, 61], DS1 would be expected to be a more potent and efficacious potentiator of native extrasynaptic δ-GABA_A_Rs than DS2. That being said, the dramatically different effects induced by DS1 and DS2 on cortical network activity can not be rationalized solely be these differences in the modulatory properties of the compounds at the recombinant receptors, and thus it could be interesting to further address the mechanism(s) underlying DS1-mediated modulation of neuoronal network activity in future studies.

The putative functional interplay between αβγ GABA_A_Rs and δ-GABA_A_Rs in the cortical networks was probed in this study using diazepam and DS1 as pharmacological tools for the respective receptor classes. Considering the above-mentioned inconclusiveness of δ-GABA_A_R expression and/or the importance of these receptors for the network activity in these cultures, any interpretations of observations made in these experiments inevitably will depend heavily on whether the network activity changes induced by DS1 are indeed mediated though δ-GABA_A_Rs or whether they arise from other yet unidentified activities of the drug. In the following, the DS1-mediated effects will be assumed to arise predominantly from δ-GABA_A_R modulation, analogously to the selective potentiation of αβγ GABA_A_R signaling exerted by diazepam.

Whereas a low degree of δ-GABA_A_R activation by DS1 was observed to reduce the sensitivity of the cortical networks to αβγ GABA_A_R signalling substantially, moderate activation of αβγ GABA_A_Rs did not seem to impact δ-GABA_A_R-mediated activity changes substantially (Figs 9 and 10). The mechanisms underlying the phenomenon of homeostatic plasticity of inhibitory neuron excitability and output are poorly understood, including how and to which extent synaptic and extrasynaptic GABA_A_Rs interact with each other to balance tonic and phasic inhibition [14]. In previous studies overexpression of α_6_βδ or α_5_βγ_2_ receptors at extrasynaptic sites in hippocampal neurons have not only been shown to result in increased tonic currents but also in significantly decreased synaptic GABA_A_R responses [72, 73]. In one of these studies, the reduction in synaptic transmission was ascribed to competition between extrasynaptic and synaptic receptors for a limited number of receptor slots at the neuron surface [73]. While this may be a plausible explanation for the observed effects in neurons with manipulated receptor expression levels, it can not be extended to this study where the putative functional interplay between the two receptor groups were assessed by pharmacological tools at neurons characterized by endogenous receptor expression levels. In this set-up, the observed differences between the concentration-response relationships for one modulator in the absence and in the presence of another modulator reflect the acute effects arising from low levels of concomitant signalling of the other receptor group. Thus, although dynamic regulation of cell surface expression levels of receptors in these neurons very well could be an underlying cause of the observed effects, other mechanisms could be involved as well, just as the homeostatic plasticity of inhibitory synapses could be rooted in multiple molecular and cellular mechanisms. For example, the phosphorylation and dephosphorylation of Ser, Thr and Tyr residues in intracellular GABA_A_R subunit regions mediated by various kinases and phosphatases has been proposed to constitute a dynamic balance modulating cell surface trafficking of GABA_A_Rs as well the functional properties of the receptors [14, 74]. Not having investigated these putative mechanisms in this study, we will refrain from further speculations about them.

## Conclusion

This work should be considered complementary to the numerous studies of synaptic αβγ GABA_A_Rs and extrasynaptic δ-GABA_A_Rs in neuronal cultures or brain slices by means of conventional electrophysiology. The network effects arising from signalling mediated by the two receptor groups were probed with several selective pharmacological tools, and the concentration-response relationships exhibited by the modulators were generally in good agreement with their functional properties at their respective recombinant target receptors. Hence, we propose that the multi-parametric analysis of MEA data allows for a detailed description of network activity changes in the cortical networks exhibited by phenotypic fingerprints determined for various GABA_A_R modulators and that this holistic approach to the study of these receptors offer interesting insights into the respective roles of αβγ GABA_A_Rs and δ-GABA_A_Rs and the putative functional interplay between them in the neurons.

## Supporting information

S1 Table The full names and the definitions of the 40 selected network activity parameters shown in Figs 2–10. (DOCX)
