# Supplementary material for: Functional characterization of GABAA receptor-mediated modulation of cortical neuron network activity in microelectrode array recordings
Source: PLoS One. 2017 Oct 13;12(10):e0186147. doi: 10.1371/journal.pone.0186147 (PMC5640229; doi:10.1371/journal.pone.0186147)
Supplement: S1 Table — (DOCX) [file pone.0186147.s001.docx]

**SI Table 1.** The full names and the definitions of the 40 selected network activity parameters shown in Figs. 2-10.

| **General Activity**  *(main activity-describing parameters)* | |
| --- | --- |
| Spike Rate | Number of spikes per second, averaged over all spike trains recorded. |
| Burst Rate | Number of bursts per minute, averaged over all units recorded. |
| Burst Period | Distance between the beginning of consecutive bursts. Burst period is equal to burst duration plus burst IBI. |
| % Spikes in Bursts | Percentage of spikes within bursts in relation to all spikes recorded within the experimental episode. |
| Event Rate | Number of events per minute. Event is defined as synchronous burst activity of at least 50% of all units in a network within a time frame of 300 ms. |
| Event Period | Distance between consecutive burst events (synchronous bursts in at least 70% of units in network within 300 ms time frame). |
| **Burst structure**  *(parameters reflect the compactness of bursting events****)*** | |
| Burst Duration | Mean lengths of bursts (ms), burst detection based on integration algorithm. |
| Burst Amplitude | Integral is defined by spike peak density in bursts and number of spikes. Burst amplitude is the peak amplitude of the integrated burst reflecting the fraction of the bursts with highest spike density. |
| Burst Area | Area under the curve after integrating the bursts, defined by burst duration, number of spikes in bursts, spike frequency in bursts. |
| Burst Spike Number | Mean number of spikes within bursts. |
| Max Spike Rate in Bursts | Maximum spike rate within a burst, computed with a binning method. |
| Burst Inter-spike- interval (ISI) | Mean time between consecutive spikes in bursts. |
| Burst Peak Frequency | Mean peak spike frequency within bursts (Hz), peak spike frequency is defined by the shortest distance (time) between two consecutive spikes in a burst. |
| Burst Spike Density | Mean frequency of spikes within bursts (Hz), defined by the average of all interspike intervals in a burst. Burst spike density increases if number of spikes in burst increases or burst duration decreases. |
| Burst Spike Rate | Mean spike rate within bursts. |
| Burst Plateau position | Burst plateau position indicates the latency from start of the burst to beginning of burst plateau. |
| **Oscillatory behavior**  *(The higher the standard deviations, the smaller the regularity of bursting events. The SD has a different bin size than its main parameter, thus describing rather the variability of the main parameter over longer temporal episodes)* | |
| Spike Rate SD | Standard deviation of number of spikes per minute, indicating the variability of spike rate within the experimental episodes. |
| Burst Rate SD | Standard deviation of number of bursts per minute, indicating the variability of burstiness of units within experimental episodes. |
| Burst Amplitude SD | Standard deviation of peak amplitude of integrated bursts. Lower values reflect consistent burst structure over recording episode, therewith a more regular/oscillatory bursting behaviour. |
| Burst Area SD | Standard deviation of number of bursts per minute, indicating the variability of burstiness of units within experimental episodes. |
| Burst Spike Number SD | Standard deviation of spike number in bursts describes the variation of single unit spike number in bursts within experimental episodes. Lower values are a measure for lower degree of variation in burst spike number, therewith more regular burst structure. |
| Burst Plateau pos. SD | Standard deviation of burst plateau position. Burst plateau position indicates the latency from start of the burst to beginning of burst plateau. Higher values of SD reflect a higher variability of burst structure within experimental episodes. |
| Max Spike Rate in Bursts SD | Standard deviation of the maximum spike rate within a burst, computed with a binning method. |
| Burst Peak Frequency SD | Standard deviation of single unit spike peak frequency in bursts. Lower values are a measure for more regularity in Burst Peak Frequency, there with a higher degree of regular burst structure within experimental episodes. |
| Burst Spike Density SD | Standard deviation of burst spike density, reflecting the variability of spike frequency in all bursts of the network within experimental episodes. |
| Event Period SD | Standard deviation of event period, reflecting the variation of single unit distances between consecutive events within experimental episodes. Lower values reflect higher population burst regularity. |
| Inter-burst-interval (IBI) SD | Standard deviation of interburst interval, reflecting the variability of burst occurrence within experimental episodes. |
| Burst Duration SD | Standard deviation of burst duration, reflecting the variability of burst duration within experimental episodes. |
| Burst Plateau pos. SD | Standard deviation of burst plateau position. Burst plateau position indicates the latency from start of the burst to beginning of burst plateau. Higher values of SD reflect a higher variability of burst structure within experimental episodes. |
| **Synchronicity**  *(The higher the CVnet values [coefficient of variation over the network], the smaller the synchronicity of spiking and bursting events)* | |
| Burst Rate CVnet | CVnet of burst rate, reflecting variation of burst rate over the network during experimental episodes. |
| Spike Rate CVnet | CVnet of spike rate, reflecting the network variability of spike rate within experimental episodes. Decrease of this parameter indicates an increase in synchronization within the network. |
| % Spikes in Bursts CVnet | CVnet of percentage of spikes in bursts, reflecting the variation of fraction of spikes within burst intervals of all spikes within experimental episode over the whole network. Decrease of this parameters reflects an increase in synchronization within the network. |
| Burst Peak Frequency CVnet | CVnet of spike peak frequency in bursts, reflecting the variation of peak frequency within experimental episodes over the whole network. Decrease of this parameters reflects an increase in synchronization within the network. |
| Burst Area CVnet | CVnet of area under the curve after integrating the bursts, defined by burst duration, number of spikes in bursts, spike frequency in bursts. The parameter describes the network variability of burst area within experimental episodes. Higher values indicate higher variability of burst structure among the network. |
| Burst Duration CVnet | CVnet of burst duration. Lower values reflect similar burst durations over the network in one recording episode, therewith a more synchronous bursting behaviour. |
| Burst Amplitude CVnet | CVnet of burst amplitude. Lower values reflect similar burst structure over the network in one recording episode, therewith a more synchronous bursting behaviour. |
| Burst ISI CVnet | CVnet of burst ISI. Lower values reflect similar burst structure over the network in one recording episode, therewith a more synchronous bursting behaviour. |
| SynAll | Burst synchronicity all: Is defined as average distance of bursts within a population burst from population burst centre. SynAll is a measure for the strength of synchronicity of a network in an inversely proportional manner. |
